# A corpus of full-text journal articles is a robust evaluation tool for revealing differences in performance of biomedical natural language processing tools

**DOI:** 10.1186/1471-2105-13-207

**Published:** 2012-08-17

**Authors:** Karin Verspoor, Kevin Bretonnel Cohen, Arrick Lanfranchi, Colin Warner, Helen L Johnson, Christophe Roeder, Jinho D Choi, Christopher Funk, Yuriy Malenkiy, Miriam Eckert, Nianwen Xue, William A Baumgartner, Michael Bada, Martha Palmer, Lawrence E Hunter

**Affiliations:** 1 Computational Bioscience Program, U. Colorado School of Medicine, 12801 E 17th Ave, Aurora, MS 8303, CO 80045, USA; 2 Department of Linguistics, University of Colorado Boulder, Boulder, 290 Hellems, CO 80309, USA; 3 Institute of Cognitive Science, University of Colorado Boulder, Boulder, MUEN PSYCH Building D414, CO 80309, USA; 4 Department of Computer Science, Brandeis University, Waltham, MS 018, MA 02454, USA

## Abstract

**Background:**

We introduce the linguistic annotation of a corpus of 97 full-text biomedical publications, known as the Colorado Richly Annotated Full Text (CRAFT) corpus. We further assess the performance of existing tools for performing sentence splitting, tokenization, syntactic parsing, and named entity recognition on this corpus.

**Results:**

Many biomedical natural language processing systems demonstrated large differences between their previously published results and their performance on the CRAFT corpus when tested with the publicly available models or rule sets. Trainable systems differed widely with respect to their ability to build high-performing models based on this data.

**Conclusions:**

The finding that some systems were able to train high-performing models based on this corpus is additional evidence, beyond high inter-annotator agreement, that the quality of the CRAFT corpus is high. The overall poor performance of various systems indicates that considerable work needs to be done to enable natural language processing systems to work well when the input is full-text journal articles. The CRAFT corpus provides a valuable resource to the biomedical natural language processing community for evaluation and training of new models for biomedical full text publications.

## Background

Text mining of the biomedical literature has gained increasing attention in recent years, as biologists are increasingly faced with a body of literature that is too large and grows too rapidly to be reviewed by single researchers [1]. Text mining has been used both to perform targeted information extraction from the literature, e.g. identifying and normalizing protein-protein interactions [2], and to assist in the analysis of high-throughput assays, e.g. to analyze relationships among genes implicated in a disease process [3]. Systems performing text mining of biomedical text generally incorporate processing tools to analyze the linguistic structure of that text. At a syntactic level, systems typically include modules that divide the texts into individual word or punctuation tokens, delimit sentences, and assign part-of-speech tags to tokens. It is becoming increasingly common to perform syntactic parsing of the texts as well, either with a full constituent parse or a dependency parse representation. At a more conceptual level, *named entity recognition*, or identification of mentions of specific types of entities such as proteins or genes, is a widely used component of systems that aim to perform entity-oriented text mining. Historically, the majority of research in biomedical natural language processing has focused on the abstracts of journal articles. However, recent years have seen numerous attempts to move into processing the bodies of journal articles. Cohen et al. [4] compared abstracts and article bodies and found that they differed in a number of respects with implications for natural language processing. They noted that these differences sometimes demonstrably affected tool performance. For example, gene mention systems trained on abstracts suffered severe performance degradations when applied to full text.

It has been previously noted that there was inadequate linguistically annotated biological text to make domain-specific retraining of natural language processing tools feasible [5]. With the release of CRAFT, we now have a large resource of appropriately annotated full text articles in the biomedical domain to enable both evaluation and retraining.

In this paper, we will introduce the linguistic annotation of a significant new resource, the Colorado Richly Annotated Full Text (CRAFT) corpus. CRAFT consists of the full contents of 97 Open Access journal articles, comprising nearly 800k tokens. CRAFT has been manually annotated with a number of elements of linguistic structure, corresponding to the functions listed above. It has also been annotated with semantic content, of biological concepts from several semantic classes characterized by biological ontologies. In prior work, we established that Open Access journal articles do not differ in terms of linguistic structure or semantic content from traditional journal articles [6] and therefore take this corpus as representative of the biomedical literature more generally. Along with this paper, we are publicly releasing 67 (70%) of the articles, constituting 70.8% of the tokens in the full corpus. It is available at http://bionlp-corpora.sourceforge.net/CRAFT/index.shtml.

The availability of the CRAFT corpus makes it possible for the first time to evaluate a number of hypotheses with exciting implications for the near-term development of biomedical text mining. In this work, we explore several uses of the CRAFT corpus for evaluating the performance of natural language processing tools. We specifically consider (a) the generalizability of training algorithms and existing models to the new corpus, and (b) the impact of the availability of full text training data for new model development. A priori, genre differences have plagued natural language processing for years, and full texts are clearly a different genre from that which most extant systems have been developed on — abstracts of journal articles [4]. Those who have worked with full text have noted various ways in which full texts differ from abstracts [7-11], mainly focusing on distributional differences of certain types of keywords and assertions. Nonetheless, a few authors have developed systems to process full text. Friedman and Rzhetsky developed the GENIES system, which processes full-text journal articles [10], Rzhetsky’s GeneWays system does as well [12], and the recent BioCreative III evaluation required systems to process full text [13].

In this work we first introduce the syntactic annotation of the CRAFT corpus. The annotation of genes and ontological concepts is described in more detail in Bada et al. (2012) [14].

Next, with this sufficiently large collection of annotated biomedical full text documents, we report the head-to-head performance of a number of language processing tools selected for their difficulty, for their relevance to any language processing task, and for their amenability to evaluation with well-annotated gold standard data. Specifically, we examined the performance of tools for: 

· Sentence boundary detection

· Tokenization

· Part-of-speech tagging

· Syntactic parsing

· Named entity recognition, specifically of gene names

Sentence boundary detection was included because it is an essential first task for any practical text mining application. Tokenization was included both because it is an essential prerequisite for any practical language processing application and because it is notoriously difficult for biomedical text (see e.g. [1,15]). Part-of-speech tagging and syntactic parsing were included because the use of syntactic analyses in biomedical text mining is a burgeoning area of interest in the field at present [16,17]. Finally, gene mention recognition was included because prior work has shown drastic differences in gene mention performance on full text across a range of gene mention systems and models [4]. We perform a broad survey of existing systems and models, and also retrain systems on the full-text data to explore the impact of the annotated training data.

Previous investigations of syntactic parser performance on biomedical text [5,18] have focused on parser performance on biomedical abstracts rather than full text publications. In particular, [18] evaluates accuracy on only 79 manually reviewed sentences, while [19,20] explore similarly small corpora of 300 and 100 sentences, respectively. The CRAFT corpus, in contrast, contains over 20,000 manually analyzed parsed sentences in the portion we are publicly releasing at this time – the full contents of 67 journal articles, containing over 500k tokens (see the Methods section for details on the partitioning of the data).

### Prior biomedical corpus annotation work

There has been significant prior work on corpus annotation in the biomedical domain. Until the very recent past, this has focused on the biological, rather than the medical, domain. The biological corpora are most relevant to the work discussed here, so we focus on them. The biomedical corpora site at http://compbio.ucdenver.edu/ccp/corpora/obtaining.shtml currently lists 26 biomedical corpora and document collections. Of this large selection, we review here only some of the most influential or recent ones.

The flagship biomedical corpus has long been the GENIA corpus [21,22]. Studies of biomedical corpus usage and design in [23,24] reviewed several biomedical corpora extant as of 2005 with respect to their design features and their usage rates outside of the labs that built them. Usage rates outside of the lab that built a corpus was taken as an indicator of the general usefulness of that corpus. These studies concluded that the most influential corpus to date was the GENIA corpus. This was attributed to two factors: the fact that this was the only corpus containing linguistic and structural annotation, and the fact that the corpus was distributed in standard, easy-to-process formats that the natural language processing community was familiar with. In contrast, the other corpora lacked linguistic and structural annotation, and were distributed in one-off, non-standard formats.

The GENETAG corpus [25] has been very useful in the gene mention recognition problem. It achieved wide currency due to its use in two BioCreative shared tasks. The BioInfer corpus [26] is a collection of 1100 sentences from abstracts of journal articles, annotated with entities according to a self-defined ontology and showing relationships between them by means of a syntactic dependency analysis. The BioScope corpus [27] is a set of 20,000 sentences that have been annotated for uncertainty, negation, and their scope. Most recently, the various data sets associated with the Association for Computational Linguistics BioNLP workshop [17,28] have been widely used for their annotations of multiple biological event types, as well as uncertainty and negation.

## Results and Discussion

### Annotation of document structure, sentence boundaries, tokens, and syntax

#### Syntactic annotation: introduction

Although CRAFT is not the first corpus of syntactically annotated biomedical text, it provides the first constituent annotation of full-text biomedical journal articles. Penn Treebank’s BioIE project provided much of the basic skeleton for the workflow of this type of annotation. However, we did have to make several new policies or expand existing PTB policies for syntactic annotation in the biomedical domain (discussed below).

The markup process of the CRAFT corpus consisted of phases of automatic parsing and manual annotation and correction of all 97 articles in the corpus. Automatic segmentation and tokenization were performed, then part of speech tags were automatically applied to every token in the data according to each token’s function in a given context (for details see below). We employed Penn Treebank’s full Part of Speech tagset (which consists of 47 tags; 35 POS tags and 12 punctuation, symbol, or currency tags) without any alterations (see Additional file 1 for the full tagset). This output was then hand corrected by human annotators.

After hand correction, the data was then automatically parsed into syntactic phrase structure trees with Penn Treebank’s phrasal tagset. Syntactic nodes indicate the type of phrase of which a token or a group of tokens is a part. They form constituents that are related to one another in a tree structure where the root of the tree encompasses the largest construction and the branches supply the relationship between the main components of the tree (subject, verb/predicate, verb arguments and modifiers) and each of these main components may contain internal phrase structure. CRAFT added 4 nodes representing article structure, CIT, TITLE, HEADING and CAPTION (discussed below), to the original tagset. The automatically processed trees were then hand corrected. Automatic parsing did not provide function tags or empty categories, which were also adapted from the Penn Treebank syntactic tagset, so those were added by hand during bracketing correction. Function tags are appended to node labels to provide additional information about the internal structure of a constituent or its role within the parent node. CRAFT added one new function tag, -FRM (discussed below). Empty categories provide a placeholder for material that has been moved from its expected position in the tree, arguments that have been dropped, such as an empty subject, or material that has been elided.

The data was finalized with two iterations of quality control verification to ensure that all the data was consistently annotated and that all policy changes that were adapted at different stages of the project were properly implemented across all data. A rough estimate of the total time required to syntactically annotate the full corpus is approximately 80 hours a week for 2.5 years (including 6 months for training).

Given the input text, “Little is known about genetic factors affecting intraocular pressure (IOP) in mice and other mammals” (PMCID 11532192), the final segmented, tokenized, part-of-speech tagged, syntactically parsed and annotated output is as follows, with each phrase in parentheses and part of speech tags to the left of their respective tokens.

(S (NP-SBJ-1 (JJ Little))

(VP (VBZ is)

(VP (VBN known)

(NP-1 (-NONE- ∗))

(PP (IN about)

(NP (NP (JJ genetic)

(NNS factors))

(VP (VBG affecting)

(NP (NP (NP (JJ intraocular)

(NN pressure))

(NP (-LRB- -LRB-)

(NN IOP)))

(-RRB- -RRB-)

(PP-LOC (IN in)

(NP (NP (NNS mice))

(CC and)

(NP (JJ other)

(NNS mammals))))))))))

.)

We describe below the major implementations and policy adaptations that yield the above tree.

#### Selection and amendment of annotation guidelines

For the POS annotation, we chose to follow the 3rd revision of the POS-tagging guidelines of the Penn Treebank project [29].

For the treebanking, we have followed the guidelines for Treebank II [30-32] and Treebank 2a [33] along with those for the BioIE project [34], which is an addendum to the Treebank II guidelines based on annotation of biomedical abstracts. Employing these guidelines of the Penn Treebank project enables us to contribute our collection of richly annotated biomedical texts to a larger collection of treebanked data that represents a multitude of genres and that already includes biomedical journal abstracts. Finally, we modified or extended these guidelines to account for biomedical phenomena not adequately addressed in them (see Additional file 2 for the CRAFT addenda to the PTB2 and BioIE guidelines). A set of these changes was made at the beginning of the project resulting from examination of the corpus articles, and further changes were made throughout the course of the project as issues arose; descriptions and examples of these changes can be seen below.

#### Training of annotators and creation of markup

The lead syntactic annotator (CW), who had five years of syntactic annotation experience, first trained the senior syntactic annotators (AL, AH), the former of whom trained a third senior syntactic annotator (TO). These lead and senior annotators were responsible for policy changes, documentation, quality control, and training of additional annotators, who were required to have some knowledge of syntax and semantics (with at least one year of completed Master’s-level linguistics coursework) and some previous experience in syntactic annotation. These additional annotators were first trained to perform POS tagging for approximately one month with Penn’s newswire training files and then on a chapter of an introductory biology book [35], followed by treebanking training for several weeks to one month on short training files obtained through the Penn Treebank project. Treebanking training continued on the aforementioned book chapter and finally on the first article of the corpus. Altogether, training for syntactic annotation lasted approximately six months. All training was performed on flat text (i.e., text that had not been automatically annotated).

For the syntactic annotation of the corpus, sentence segmentation, tokenization, and POS markup was first automatically generated using the GENIA parser. Each article’s automatically generated markup was manually corrected by one annotator in the lex mode of Emacs. This was followed by the automatically generated treebanking of these articles (with the corrected segmentation, tokenization, and POS markup) using the parser of the OpenNLP project. Each article’s automatically generated treebanking markup was then manually corrected by one annotator using TreeEditor. Since they are not generated by this parser, the annotators used TreeEditor to add empty categories, which are syntactic place holders in the tree construction that indicate arguments that have been moved from their expected positions in the trees, and functions tags, which specify additional information about phrases not represented in the treebanking markup, e.g., the location of an action. Additionally, sentence-segmentation errors not previously found were corrected manually outside of TreeEditor, as it does not have the capability of merging sentences. The corrected output of this annotator was checked by the syntactic lead annotator.

The output of the syntactic lead then underwent the final phase of syntactic annotation, referred to as the quality-control phase. This phase consisted of automatic validation of POS tags (e.g., checking that a phrase annotated as a prepositional phrase actually begins with a word annotated as a preposition) and of sentences (e.g., checking that each S node had a subject and a verb at the appropriate level of attachment) using CorpusSearch followed by manual correction of indicated errors. This step allowed us to confirm tree uniformity, to verify that errors had not been introduced during the manual correction of previous passes, and to ensure that changes in annotation guidelines or policy made during the project were consistently reflected in the final output. For example, during the course of annotation, the treatment of prepositional phrases beginning with “due to” changed from being annotated as recursive prepositions, i.e., (PP due (PP to)), to being annotated as flat multiword prepositions, i.e., (PP due to). A validation script was written to detect recursively annotated occurrences of such prepositional phrases, an example of which is provided below.

These results explain why defective PDGF signal transduction results in a reduction of the v / p cell lineage and ultimately in perinatal lethality due to vessel instability (Hellström et al. 2001).68 PP-PRP: 68 PP-PRP, 69 IN, 70 due, 71 PP(62 NP (63 NP (64 JJ perinatal) (66 NN lethality))(68 PP-PRP (69 IN due)

(71 PP (72 IN to)

(74 NP (75 NN vessel) (77 NN instability)))))))

This error message indicates that there is a recursive PP error and provides the full sentence, the reference number(s) of the element(s) involved in the error, and the current parse of the tree. Given this output, the annotator manually corrected this error in the file by deleting the extra PP node for “to”.

#### Guidelines

Full-text journal articles present issues that can be uniquely distinguished from the style of the abstracts that the Penn BioIE project annotated. We found that Penn’s guidelines for biomedical abstract annotation did not cover the increased technical complexity of a full-length article, such as the parenthetical information, definitions, and figure and table captions found throughout a full-text article, necessitating regular policy review and addendum construction. Major changes to Penn’s guidelines include addition of node labels TITLE, HEADING and CAPTION to replace the -HLN function tag (see below), and CIT for citations. We have added one new function tag, -FRM, to the top-level constituent (S) of formulas, where a mathematical symbol (<, >, =) is treated as a verb. The use of the PRN node label has been expanded from the TB2a policy [33], which only allows for a clausal PRN (reference, appositive-like adjectives). Because of the large number of nominals and other parentheticals in the CRAFT data we have allowed any node label inside of PRN. The use of the -TTL function tag has been slightly modified from ETTB as well. Each of these node and function label additions and expansions have been made in order to provide labeling that accurately represents the more complex structure of biomedical articles.

We have also changed how shared adjuncts are bracketed, which are now adjoined to coordinated VP or S, added more structure to single token coordinated NMLs, and refined Penn’s POS and tokenization policy to account for additional symbols, such as ∘ (degree) (as in 35°C). Another significant change we have made is the elimination of PP-CLR. PTB2 allows for PP-CLR on verbal arguments. However, we felt that this policy was not clearly defined and it was difficult to consistently apply. We have retained the -CLR in S-CLR for resultatives and secondary predicates.

The last change we implemented was the complete elimination of the empty category *P* (placeholder for distributed material) introduced in the Penn BioIE guideline addendum. With the increased complexity of full-length articles, we felt that these policies were difficult to apply consistently and greatly increased the complexity of the annotation and resulting trees. We maintain that existing policy on NML and NP coordination preserves much of the same information represented by *P*.

In PTB2, the -HLN function tag indicates a headline or a dateline, as found in newswire texts. However, the section headings in journal articles have a slightly different function and convey different information than a news headline. Since the treebanked data are journal articles, we are using more informative labels for nodes that would have been tagged with -HLN (see example below) based on newswire bracketing guidelines (Guidelines [31] Section Ongoing and future work).

(FRAG-HLN (NP-SBJ-1 Clinton)

(S-PRD (NP-SBJ ∗*PRO*∗-1)

(VP to leave)))

‘‘Clinton to leave'' (BioIE addendum)(FRAG-HLN (NP-SBJ-1 3 soldiers)

(VP killed (NP ∗−1)

(PP by

(NP-LGS bomb))))

‘‘soldiers killed by bomb'' (BioIE addendum)

#### CRAFT addenda

Labels were created for information that is unique to the structuring of a journal article. The CRAFT annotation guidelines add TITLE, HEADING, and CAPTION node labels to denote these sections of journal articles. Below are several examples of usage. (From CRAFT addendum to PTB2 and BioIE guidelines, see Additional file 2)

*Journal title: TITLE*(TITLE (NP PLoS

Genetics))

*Section headings: HEADING*(HEADING (NP Abstract))(HEADING (NP Introduction))(HEADING (NP Results))*Figure, Table, and Picture Captions: CAPTION*(HEADING (NP Figures and Tables))(HEADING (NP Figure 1))(CAPTION (NP (NP An

(QP almost two-fold)

range)

(PP of

(NP IOP))

(PP between

(NP (ADJP genetically distinct)

mouse strains))

.))‘‘An almost two-fold range of IOP between genetically distinct mouse strains.''(PMCID 1152192)(TITLE (FRAG (NP (NP Intraocular pressure)

(PP in

(NP (ADJP genetically distinct)

mice)))

:

(NP an

(NML (NML update)

(CC and)

(NML strain survey)))))

‘‘Intraocular pressure in genetically distinct mice : an update and strain survey''(PMCID 11532192)

These nodes require internal structure the same as other main text nodes. However, TITLE, HEADING, and CAPTION nodes have only one daughter. In cases where titles, headings or captions are not complete sentences, FRAG may be used to make a single constituent of the daughter nodes.

(TITLE (FRAG (NP Complex Trait Analysis of the Mouse Striatum)

:

(S Independent QTLs Modulate Volume and Number)))

While TITLE, HEADING, and CAPTION are new nodes that have been added to PTB2’s original tag set, we have also changed the scope of some existing node labels, such as PRN (from CRAFT addendum to PTB II and BioIE guidelines).

#### PRN and CIT

We have expanded the use of PRN to include citations that consist of other referential material such as page or footnote numbers, figure and table information, or extra-sentential details. The PRN node is put inside of whichever node it seems to be modifying. Sentence-final parentheticals modifying the entire sentence are placed inside the VP containing the matrix verb, mirroring the placement of sentence-level adjuncts.

#### Adding primary label CIT for inline citations

In Penn BioIE Addendum ([34] Section 7.2), citations are annotated as follows:

(PRN -LRB-

(FRAG Shelton et al., 1983)

-RRB- ))

Since citations are pervasive in journal articles and books, we have added a CIT node for inline citations. The internal structures for citations are flat. CIT applies only to author references that occur inside of parentheses.

(CIT -LRB- Shelton et al., 1983 -RRB-)

(CIT -LRB- A. - K.H. and V.E. Papaioannou , unpublished observations -RRB-)‘‘(A. - K.H. and V.E. Papaioannou, unpublished observations)'' (PMCID 12079497)

All other, non-parenthetical references are bracketed as normal text.

Citations that are part of the argument structure of a larger sentence are annotated fully as ordinary text:

(NP (NP The second paper)

,

(PP by

(NP (NP Davies)

(ADVP-ETC et al))))

‘‘The second paper, by Davies et al'' (PMCID 11597317)(VP reviewed

(NP-1 ∗)

(PP in (NP (NP (NP Furumura)

(ADVP-ETC et al.))

(NP-TMP 1996))))

‘‘reviewed in Furumura et al. 1996'' (PMCID 14737183)

#### Expansion of PRN

We have expanded its use to include citations that consist of other referential material such as page or footnote numbers, figure and table information, or extra-sentential details. If the sentence contains only one parenthetical at the end of the sentence, then this is a daughter of the VP; otherwise, it is within whichever node it seems to be modifying.

(S (NP-SBJ These mutations

(VP shift

(NP (NP the spectral profiles

(PP of

(NP the translation products))))

(PRN [

(NP 4 , 11)

]))) .)

‘‘These mutations shift the spectral profiles of the translation products [4,11].''(PMCID 12079497)(S (NP-SBJ-1 R1 ES cells

(PRN [

(NP 20)

]))

(VP were

(VP maintained

(NP-1 ∗)

(PP under

(NP standard culture conditions ))

(PP in

(NP (NP the presence)

(PP of

(NP LIF)))))) .)

‘‘ES cells [ 20 ] were maintained under standard culture conditions in the presence of LIF..'' (PMCID 12079497)

#### Addition of -FRM function tag

We have added one new function tag, -FRM, to the top-level constituent (S) of formulas in which a mathematical symbol (<, >, =) is treated as a verb.

(S-FRM (NP-SBJ (NN p))

(VP <

(NP 0.05))

‘‘p < 0.05''

The above is interpreted as “p is less than 0.05.” Orthographically, the copula is not realized—thus we have created the -FRM tag to denote the difference between formulas and canonical sentence structure.

#### Shared VP and S adjuncts

In the PTB, shared adjuncts for coordinated VPs are left at the conjunction level:

(S (NP-SBJ-1 the company)

(VP expects

(S (NP-SBJ-1 ∗*PRO*∗)

(VP to

(VP (VP obtain

(NP regulatory approval))

and

(VP complete

(NP transaction))

(PP by

(NP year-end)))))))

‘‘The company expects to obtain regulatory approval and complete transaction by year-end.'' (Penn BioIE guidelines)“by year-end” is shared by both VPs “obtain regulatory approval” and “complete transaction”, but is attached at the same level as those two VPs to form a flat structure. CRAFT adds a layer of VP so that the PP modifier and the coordinated VP are at different levels of attachment to make more explicit the shared distribution of the PP. (S (NP-SBJ-1 the company)

(VP expects

(S (NP-SBJ-1 PRO∗)

(VP to

(VP (VP (VP obtain

(NP regulatory approval))

and

(VP complete

(NP transaction))

(PP by

(NP year-end)))))))

Shared modifiers can also occur at the S level. When two clauses share a modifier, the modifier is adjoined to the coordinated S’s. (S (PP-TMP After

(NP (NP puncture)

(PP of

(NP (NP coagulated blood)

(PP from

(NP the corpora cavernosa)))) ))

(S (S (NP-SBJ urine retention)

(VP developed))

and

(S (NP-SBJ-1 a suprapubic catheter)

(VP had

(S (NP-SBJ-1 ∗)

(VP to

(VP be

(VP introduced

(NP-1 ∗)

(ADVP temporarily)

(PP-PRP for

(NP urine drainage) ))))))))

.)

‘‘After puncture of coagulated blood from the corpora cavernosa urine retention developed and a suprapubic catheter had to be introduced temporarily for urine drainage.'' (BioIE addendum section 11.10).

#### Tokenization and Part of Speech tagging of hyphens and symbols

We adopted Penn’s tokenization policy regarding hyphens, slashes, dashes and symbols, in which expressions containing hyphens and symbols are split into multiple tokens, with the exception of a list of bound affixes that don’t provide meaning in isolation (see Penn’s single token hyphenated words list in the Additional files).

#### Hyphens, slashes, and dashes

Dashes are split and are interpreted as prepositional phrases when they are used to denote a range, as in pages in a parenthetical reference:

(PRN (-LRB- [)

(NP (NP (CD 13))

(PP (SYM -)

(NP (CD 19))))

(-RRB- ]))

‘‘We have focused on developing the mouse system for IOP and glaucoma studies [13-19].'' (PMCID 11532192)

In the above citation the dash is read as “to.” When a dash is pronounced (‘to’, ‘negative,’ or ‘minus’) it is tagged SYM to distinguish hyphens and dashes that perform syntactic functions from those that simply link words together.

The negative symbol is also annotated as a pronounced symbol and receives the SYM POS tag:

(NP (NP (NN average))

(PP (IN of)

(NP (QP (SYM -) (CD 0.3))

(NN mmHg))))

‘‘average of -0.3 mmHg''

We also split off all slashes in the text into separate tokens. When a slash is pronounced (typically ‘of’ or ‘per’) it received the SYM POS tag:

(NP (CD 100) (NNS mg))

(PP (SYM /)

(NP (NN kg))))

‘‘100 mg/kg''

Compare the above with use of HYPH for hyphens and slashes that are not pronounced:

(NP (NML (NN neuron) (HYPH -) (VBG packing))

(NN density))

‘‘neuron-packing density''(NP (NML (NN BALB) (HYPH /) (NN cJ))

(NN strain))

‘‘BALB / cJ strain''

In keeping with Penn’s tokenization policy we decided to treat numbers in temperatures as multi-token expressions. Temperatures containing a ‘°’ (degree) symbol are split into two tokens; the number is POS tagged as a cardinal number, CD, and ‘°C’ forms a constituent that is POS tagged as a singular noun as in “37°C,” (NP(CD 37)(NN °C)).

The percent symbol is also split. However, it receives the POS tag NN for singular noun, rather than SYM.

(NP (NML (QP (JJR greater) (IN than) (CD 90))

(NN %))

(NN inhibition))

‘‘greater than 90% inhibition'' (PMCID 11532192)(NP (NML (CD 4) (NN%))

(NN fat))

‘‘4% fat'' (PMCID 11532192)

Symbols in mathematical formulas are split and POS tagged as SYM.

(S-FRM (NP-SBJ (NN n))

(VP (SYM =)

(NP (CD 4))))

‘‘n = 4''(S-FRM (NP-SBJ (NN P))

(VP (SYM <)

(NP (CD 0.0001))

‘‘P < 0.0001''

We did not split certain symbols from their names, since they are part of the name as a whole and do not serve a specific function such as joining terms together (hyphen) or providing other syntactic or semantic information (such as ’<’ interpreted as a verb or ’/’ interpreted as a preposition).

(NP (JJ homozygous) (JJ lethal)

(PRN (-LRB- -LRB-)

(NP (NN ‡ XPCS))

(-RRB- -RRB-)))

‘‘homozygous lethal (‡XPCS)'' (PMCID 17020410)(NP The (NML p53 δ PGFP exchange)

(NN construct))

‘‘The p53 δ PGFP exchange construct'' (PMCID 16870721)

#### Elimination of -CLR

In PTB2 one use of the function tag -CLR is to label prepositional phrases that have a closer relationship with a verb than simply that of an adjunct. Whether a PP is an argument or not to a large extent depends on the specific verb and it is hard to make a general characterization about the nature of this category without referring to this verb. Therefore the Penn BioIE Addendum has a long list of verbs that take a PP that can be labeled -CLR. Below is an example of PP tagged -CLR:

(VP account

(PP-CLR for

(NP her disappearance)))

We believe such argument structure information is better handled in a separate layer of Propbank-style annotation that focuses on the argument structure of each verb. In the treebank annotation, we avoid using this functional tag. Following CRAFT’s policy, the above example is annotated as follows:

(VP account

(PP for

(NP her disappearance)))

Note that we have retained the use of S-CLR to mark resultatives and secondary predicates, as defined in the Treebank 2a guidelines:

(S (NP-SBJ uncertainty)

(VP drives

(NP-3 people)

(S-CLR (NP-SBJ ∗ PRO ∗-3)

(ADJP-PRD wild))))

#### Modification of -TTL

The -TTL function tag was originally used only to mark the titles of created works. However, it also marks a form of nominalization, as titles whose internal structure is not nominal can behave nominally. For example, “In the Heat of the Night” or “One Flew Over the Cuckoo’s Nest” can function as the subject of a sentence or the object of a preposition.

The following is an example of -TTL in its more traditional usage:

(NP (NP (NP the EU project)

(NP EUMORPHIA)))

,

‘‘

(S-NOM-TTL (NP-SBJ (-NONE- ∗ PRO∗))

·

(VP (VBG Understanding)

(NP (JJ human) (JJ molecular)

(NML physiology and pathology))

(PP-MNR through

(NP (NP integrated functional genomics)

(PP-LOC in

(NP the mouse model))) )))

''))

‘‘the EU project EUMORPHIA, ‘‘Understanding human molecular physiology and pathology through integrated functional genomics in the mouse model'' '' (PMCID 15345036)

As in ETTB, CRAFT allows the extension of -TTL to other instances of this referential nominalization that can occur outside of titles. For example:

(S (S (NP-SBJ-1 Significance)

(VP was

(VP set (NP-1 ∗)

(PP at

(S-TTL-FRM (NP-SBJ p)

(VP <

(NP 0.05)) )))))

and

(S (ADJP-TTL-SBJ suggestive)

(VP refers

(PP to

(S-TTL-FRM (NP-SBJ p)

(VP <

(NP 0.63)) ))))

.)

‘‘Significance was set at p < 0.05 and suggestive refers to p < 0.63.'' (PMCID 15938754)

This sentence has two nominalizations. *Suggestive* refers to the word *suggestive* itself rather than the quality of being suggestive. Similarly, the formula (analyzed internally as S because it is read aloud as “P is less than 0.63”) is functioning nominally within the larger context of the sentence, in its position as object of a preposition.

#### Elimination of *P*

*P* is used in the Penn BioIE project as a place-holder for a distributed premodifier or head, and it is used exclusively in coordinated nominal constructions. Here is an example where *P* is used in the Penn BioIE project:

(NP (NP K-

(NML-1 ∗ P ∗))

and

(NP N-

NML-1 ras)))

CRAFT annotates the tree as: (NP (NML K- and N-) ras)

This structure would represent the fact that *K-* and *N-* are both modifiers of *ras*. Please refer to the CRAFT addendum to PTB2 and Penn BioIE guidelines for a more detailed discussion of *P*.

#### NML Modification

In general, we have maintained the current policies of PTB2a regarding annotation within NP: *P* is not used and the NML node label is used for sub-NP nominal substrings (see section 12.2.1 of [34] Penn BioIE addendum for detailed information about NML). However, in conjunction with other policy changes that explicitly annotate the scope of coordinated structures (see section *Shared VP and S adjuncts*, above), we have slightly expanded the use of NML in certain single-token coordinated structures. By current TB2a policy, single-token coordinated nominal heads with shared premodifiers are left flat:

(NP the cats and dogs)

In CRAFT, we explicitly show the scope of *the*, which is modifying both *cats* and *dogs*, by putting a NML node around *cats and dogs*:

(NP the

(NML cats and dogs))

More examples of this expanded use of NLM from PMCID 11532192: (PP at

(NP each

(NML dose and time)))

(NP the

(NML species, strain and environment))

(PP in

(NP (NML early onset)

(NML obesity and diabetes)))

In this way, we more closely align the annotation of these single-token coordinated heads with existing PTB2a policy regarding the use of NML in multi-token coordinated phrases with shared premodifiers:

(NP the

(NML (NML grey cats)

and

(NML brown dogs)))

(NP the

(NML (NML pupil)

and

(NML optic nerve)))

‘‘the pupil and optic nerve'' (PMCID 11532192)

As in PTB2a, we also use NML in multi-token nominal premodifiers of nouns, as in:

(NP (NML red blood cell)

destruction)

‘‘red blood cell destruction'' (PMCID 12925238)…where *red* and *blood* modify *cell*, forming the sub-NP constituent *red blood cell*, which modifies *destruction*.

Some other examples of NML in CRAFT marking this type of complex nominal modifier:

(NP the overall

(NML gene expression)

profiles)

‘‘the overall gene expression profiles'' (PMCID 16504174)(NP (NML (NML C57BL / 6J)

and

(NML 129x1 / SvJ))

inbred strains)

‘‘C57BL/6J and 129x1/SvJ inbred strains'' (PMCID 17590087)(NP a

(NML (QP greater than 90)

%)

inhibition)

‘‘a greater than 90% inhibition'' (PMCID 11532192)

#### Annotation products and quality assessment

The syntactic annotation of the CRAFT corpus consisted of manual annotation, including manual correction of automatic parsing, of 97 full-text biomedical journal articles. The 97 fully syntactically annotated articles yielded 30,800 sentences and 793,627 tokens, and include 619,567 syntactic nodes, 101,022 function tags, and 37,178 empty categories. The initial release of 67 articles yielded 21,710 sentences and 561,020 tokens and include 437,250 syntactic nodes, 71,522 function tags, and 25,978 empty categories. See Additional file 3 for counts of each node, tag, and empty category.

When the OpenNLP syntactic parser output was compared to the gold standard it achieved 67.46 recall and 33.68 precision, whereas the average accuracy of annotators when compared to the gold standard is 94.67 recall and 94.94 precision (see Methods, Inter-annotator agreement section for a description of IAA calculations). This indicates a large human annotation effort to correct automatic output. Automatic parsing of biomedical literature is not consistent enough to rely only on automatic methods to provide precise data. Average inter-annotator agreement is 90.91 recall and 90.30 precision. Full details of IAA are available in Table 1.

**Table 1:** Inter-annotator agreement of syntactic annotation of the CRAFT corpus

	**Annotator-Annotator IAA**			**Gold-Annotator IAA**			**Gold-Parser IAA**
	**A1-A2**	**A1-A3**	**A2-A3**	**A1**	**A2**	**A3**	
Recall	91.02	92.31	89.39	95.92	94.98	93.16	67.46
Precision	90.58	90.18	90.13	94.98	94.58	94.39	33.68

### Evaluation of named entity recognition systems and syntactic parsers

We consider the performance of existing systems on several tasks important for biomedical natural language processing: gene/protein mention recognition and syntactic analysis of text, including the syntactic pre-processing steps of sentence boundary detection, tokenization, and part of speech tagging.

For each tool assessed, we produced results over the CRAFT text using the models with which they are distributed and compared the produced annotations to the CRAFT gold standard annotations using standard measures. We also retrained several of the tools on the CRAFT data to assess the impact of retraining, performing five-fold cross-validation of a training set sub-selected from CRAFT. We report the performance on a held-out development set in both cases (see Methods Section Data partitioning).

#### Gene mention recognition

The CRAFT corpus semantic annotations include annotation of gene mentions. The Sequence Ontology [37] was used as the target ontology for mentions of sequences, including genes and gene products. Entrez Gene [38,39] identifiers are used to associate gene mentions with a specific gene. We utilized these annotations to evaluate several popular named entity recognition (NER) systems that focus on recognition of protein or gene names. NER of gene/protein mentions has been the subject of several shared tasks [40] and is a common step in other BioNLP applications, such as protein-protein interaction extraction or gene-disease relation extraction. NER systems aim to identify relevant names in text, and delimit the boundaries of those names. They do not typically attempt to map those names to a specific database identifier (this is a separate task referred to as Gene Normalization [41]) and therefore our evaluation focuses only on the detection of relevant strings in the text.

Bada et al. describes the semantic annotation of CRAFT [14]. The annotation identifies mentions of genes and their products, including a determination of type (e.g. the Sequence Ontology concepts of “gene” or “polypeptide”). We compared these annotations with mentions found by the NER systems. The sequence type annotations, however, are very detailed and the set of annotations for a single type do not in every case correspond to a cohesive set of annotation categories from a given NER system/model, such as the “protein” category of the BioCreative datasets [40,42], or NLPBA’s “DNA” and “RNA” categories [43]. The problem of inconsistency among annotation category sets has also been investigated by [44], who introduced an aggregate tag, gene-or-gene-product (GGP).

To achieve better coverage, we also aggregated several semantic classes utilized in CRAFT based on domain knowledge, for the purpose of evaluation. The mappings are listed in Table 2. The names reflect a combination of the main focus of the class and the Kleene star (‘*’) character used in regular expressions. In the final form, the aggregations are explicitly defined in terms of specific classes and do not make use of regular expressions (see Additional file 4 for the complete set of aggregations). We tested various combinations of NER system categories to CRAFT semantic classes for each system, depending on the categories used by the system. In the tables below, we use a lowercase descriptor for the source NER system categories and an all caps descriptor for the target CRAFT semantic classes. For instance, Table 3 refers to “protein-POLYSTAR”. This means that the ABNER category of “protein” was allowed to match any of the CRAFT classes listed for POLYSTAR in Table 2, including “polypeptide”, “macromolecular complex”, or “transcript”. That is, if ABNER produced an annotation with the category “protein” where the CRAFT gold standard has e.g. an annotation with the class “transcript”, this was counted as a true positive in the evaluation.

**Table 2:** Semantic class groupings for CRAFT

**Mapping strategy**	**Source Semantic class**
STAR	(any class)
GENE	gene
POLY	polypeptide
	QTL
	cDNA
	gene
GENESTAR	gene or polypeptide
	gene or polypeptide or macromolecular complex
	gene or transcript or polypeptide
	gene or transcript or macromolecular complex
	macromolecular complex
	polypeptide
	polypeptide or macromolecular complex
POLYSTAR	promoter
	transcript
	transcript or polypeptide
	transcript or polypeptide or macromolecular complex

**Table 3:** Precision/Recall/F1-score results for gene mention detection over CRAFT development set: ABNER with distributed model trained on BioCreative I using different evaluation mapping strategies

	**ABNER BioCreative** **protein-STAR**			**ABNER BioCreative** **protein-GENE**			**ABNER BioCreative** **protein-POLYSTAR**		
	**Prec**	**Recall**	**F1**	**Prec**	**Recall**	**F1**	**Prec**	**Recall**	**F1**
strict	0.35	0.46	0.40	0.12	0.31	0.18	0.20	0.62	0.30
overlap	0.50	0.69	0.58	0.23	0.64	0.34	0.23	0.74	0.35
shared	0.49	0.65	0.56	0.22	0.57	0.32	0.23	0.73	0.35
subspan	0.50	0.69	0.58	0.23	0.64	0.34	0.23	0.74	0.35

Tables 3, 4, 5, 6, 7, 8, 9, 10, 11 and 12 show the results for the gene mention systems with the distributed models. Distributed models are trained on one of several available gene mention corpora: the NLPBA corpus [43], the GENIA corpus [22], the BioCreative I gene mention corpus [42], or the BioCreative II gene mention corpus [40]. Two sets of results are provided, based on a comparison of the system output on (a) the development portion of the CRAFT public release data set and (b) the complete initial public release of CRAFT of 67 files (see Methods section Data partitioning). We performed experiments with several variables: 

·**System:** The system used to produce the results.

·**Model:** The specific model used by the system to produce the results.

·**Annotation comparison strategy:** The specific strategy used in assessing precision and recall of gold standard annotations (see Table 13).

·**Annotation class mapping strategy:** The defined mappings from annotation classes in the source system model to the CRAFT model (see Table 2).

**Table 4:** Precision/Recall/F1-score results for gene mention detection over CRAFT initial release: ABNER with distributed model trained on BioCreative I using different evaluation mapping strategies

	**ABNER BioCreative** **protein-STAR**			**ABNER BioCreative** **protein-GENE**			**ABNER BioCreative** **protein-POLYSTAR**		
	**Prec**	**Recall**	**F1**	**Prec**	**Recall**	**F1**	**Prec**	**Recall**	**F1**
strict	0.32	0.36	0.34	0.11	0.29	0.16	0.16	0.41	0.23
overlap	0.48	0.55	0.51	0.19	0.52	0.28	0.21	0.57	0.31
shared	0.46	0.53	0.50	0.18	0.50	0.27	0.21	0.55	0.30
subspan	0.47	0.55	0.51	0.19	0.52	0.28	0.21	0.57	0.31

**Table 5:** Precision/Recall/F1-score results for gene mention over CRAFT development set: ABNER with distributed model trained on NLPBA using indicated evaluation mapping strategies

	**ABNER NLPBA** **protein-STAR**			**ABNER NLPBA** **star-POLYSTAR**			**ABNER NLPBA** **protein-GENESTAR**		
	**Prec**	**Recall**	**F1**	**Prec**	**Recall**	**F1**	**Prec**	**Recall**	**F1**
strict	0.38	0.44	0.41	0.15	0.58	0.24	0.15	0.33	0.21
overlap	0.47	0.55	0.51	0.17	0.69	0.28	0.21	0.46	0.29
shared	0.46	0.54	0.50	0.17	0.67	0.27	0.21	0.45	0.29
subspan	0.47	0.55	0.51	0.17	0.69	0.28	0.21	0.46	0.29

**Table 6:** Precision/Recall/F1-score results for gene mention over CRAFT initial release set: ABNER with distributed model trained on NLPBA using indicated evaluation mapping strategies

	**ABNER NLPBA** **protein-STAR**			**ABNER NLPBA** **star-POLYSTAR**			**ABNER NLPBA** **protein-GENESTAR**		
	**Prec**	**Recall**	**F1**	**Prec**	**Recall**	**F1**	**Prec**	**Recall**	**F1**
strict	0.30	0.34	0.32	0.11	0.41	0.17	0.13	0.29	0.18
overlap	0.39	0.44	0.41	0.14	0.57	0.23	0.15	0.36	0.22
shared	0.38	0.42	0.40	0.14	0.54	0.22	0.15	0.36	0.21
subspan	0.38	0.43	0.41	0.14	0.57	0.23	0.15	0.36	0.22

**Table 7:** Precision/Recall/F1-score results for gene mention over CRAFT development set: BANNER with distributed model trained on BioCreative II using indicated evaluation mapping strategies

	**BANNER BioCreative** **protein-STAR**			**BANNER BioCreative** **protein-GENESTAR**			**BANNER BioCreative** **protein-POLYSTAR**		
	**Prec**	**Recall**	**F1**	**Prec**	**Recall**	**F1**	**Prec**	**Recall**	**F1**
strict	0.38	0.61	0.47	0.16	0.49	0.25	0.20	0.78	0.32
overlap	0.49	0.80	0.61	0.25	0.77	0.38	0.22	0.85	0.35
shared	0.49	0.79	0.60	0.25	0.75	0.37	0.22	0.85	0.35
subspan	0.49	0.80	0.61	0.25	0.76	0.38	0.22	0.85	0.35

**Table 8:** Precision/Recall/F1-score results for gene mention over CRAFT initial release: BANNER with distributed model trained on BioCreative II using indicated evaluation mapping strategies

	**BANNER BioCreative** **protein-STAR**			**BANNER BioCreative** **protein-GENESTAR**			**BANNER BioCreative** **protein-POLYSTAR**		
	**Prec**	**Recall**	**F1**	**Prec**	**Recall**	**F1**	**Prec**	**Recall**	**F1**
strict	0.35	0.51	0.41	0.14	0.42	0.21	0.18	0.60	0.28
overlap	0.46	0.69	0.56	0.20	0.63	0.30	0.22	0.76	0.34
shared	0.46	0.68	0.55	0.20	0.61	0.30	0.22	0.74	0.34
subspan	0.46	0.69	0.56	0.20	0.63	0.30	0.22	0.75	0.34

**Table 9:** Precision/Recall/F1-score results for gene mention over CRAFT development set: LingPipe with distributed model trained on Genia using indicated evaluation mapping strategies

	**LingPipe Genia** **proteinstar-STAR**			**LingPipe Genia** **proteinstar-GENESTAR**			**LingPipe Genia** **protein-STAR**		
	**Prec**	**Recall**	**F1**	**Prec**	**Recall**	**F1**	**Prec**	**Recall**	**F1**
strict	0.29	0.38	0.33	0.10	0.25	0.14	0.30	0.37	0.33
shared	0.35	0.47	0.40	0.14	0.34	0.20	0.36	0.45	0.40
subspan	0.36	0.48	0.41	0.14	0.36	0.20	0.37	0.47	0.41
overlap	0.36	0.48	0.41	0.14	0.36	0.20	0.37	0.47	0.41

**Table 10:** Precision/Recall/F1-score results for gene mention over CRAFT initial release set: LingPipe with distributed model trained on Genia using indicated evaluation mapping strategies

	**LingPipe Genia** **proteinstar-STAR**			**LingPipe Genia** **proteinstar-GENESTAR**			**LingPipe Genia** **protein-STAR**		
	**Prec**	**Recall**	**F1**	**Prec**	**Recall**	**F1**	**Prec**	**Recall**	**F1**
strict	0.21	0.31	0.25	0.07	0.22	0.11	0.22	0.31	0.26
shared	0.27	0.41	0.33	0.09	0.28	0.14	0.22	0.31	0.26
subspan	0.28	0.42	0.33	0.09	0.29	0.14	0.29	0.41	0.34
overlap	0.28	0.42	0.33	0.09	0.29	0.14	0.29	0.41	0.34

**Table 11:** Precision/Recall/F1-score results for gene mention over CRAFT development set: LingPipe with distributed model trained on GeneTag using indicated evaluation mapping strategies

	**LingPipe GeneTag** **gene-STAR**			**LingPipe GeneTag** **gene-GENE**			**LingPipe GeneTag** **gene-GENESTAR**		
	**Prec**	**Recall**	**F1**	**Prec**	**Recall**	**F1**	**Prec**	**Recall**	**F1**
strict	0.26	0.69	0.38	0.12	0.60	0.20	0.12	0.61	0.20
shared	0.31	0.83	0.45	0.15	0.80	0.26	0.16	0.79	0.26
subspan	0.32	0.86	0.46	0.16	0.85	0.27	0.16	0.85	0.27
overlap	0.32	0.86	0.46	0.16	0.85	0.27	0.16	0.85	0.27

**Table 12:** Precision/Recall/F1-score results for gene mention over CRAFT initial release set: LingPipe with distributed model trained on GeneTag using indicated evaluation mapping strategies

	**LingPipe GeneTag** **gene-STAR**			**LingPipe GeneTag** **gene-GENE**			**LingPipe GeneTag** **gene-GENESTAR**		
	**Prec**	**Recall**	**F1**	**Prec**	**Recall**	**F1**	**Prec**	**Recall**	**F1**
strict	0.22	0.63	0.33	0.08	0.56	0.15	0.10	0.58	0.17
shared	0.30	0.85	0.44	0.12	0.84	0.22	0.14	0.84	0.24
subspan	0.30	0.86	0.45	0.13	0.86	0.22	0.14	0.86	0.24
overlap	0.30	0.86	0.45	0.13	0.87	0.22	0.14	0.87	0.25

**Table 13:** Annotation comparison strategies

**Strict**	Requiring matches at both the left and right edges of the name span
**Overlap**	Allowing any degree of overlap between the system-identified name span and the gold standard name span
**Shared**	Requiring a match only at one of the left or right edge of the name span
**Subspan**	Subsumption, where the boundaries of the system-identified name are within the span of the gold standard annotation, or vice versa

In performing annotation comparison, gene mentions were scored with respect to four progressively less strict types of mention boundaries due to differences in what the different automatic taggers considered proper mention boundaries. The various strategies are summarized in Table 13.

The specific results shown in Tables 3, 4, 5, 6, 7, 8, 9, 10, 11 and 12 represent only a few of the possible semantic class mappings. While full results are available as additional material (see Additional files 5 and 6), here we have selected the 3 top-performing mappings for each system/model combination. Examination of the tables shows significant variability in performance, depending on the different variable settings. Some variability can likely be attributed to differences in size or quality of the underlying training corpora (we note that the reported results for the ABNER system trained on NLPBA is worse than for the same system trained on the BioCreative data), while some likely has to do with differences in the learning algorithms. With regard to overall system performance, the BANNER system produced consistently higher results than other systems with various semantic class mappings, though it was only tested with one distributed model.

A comparison with the ABNER system, also using a BioCreative-derived model, shows that BANNER outperforms the ABNER BioCreative model. ABNER with the BioCreative model outperforms the same system with the NLPBA model; this indicates that the BioCreative gene mention data is more similar to CRAFT gene annotations than the NLPBA data. In general, increasing the scope of the semantic classes considered to be a gene mention in CRAFT increases precision. This indicates that most gene NER systems employ a generous definition of a “gene”, while the CRAFT annotations are more fine-grained and semantically precise. Finally, we note that the results of all systems and models are consistently worse on the full CRAFT initial release set than the smaller development set. This suggests that despite our best efforts to partition the CRAFT data into unbiased subsets, there may still be some important variation.

For those NER systems that were straightforwardly trainable, we retrained them on the CRAFT data (see Methods, Section NER Tools for details). Tables 14 and 15 show the performance of the retrained GM systems on the development portion of the CRAFT corpus. The retrained systems are trained with the aggregations of the semantic classes derived from the Sequence Ontology, shown in column 2 of Table 2. The systems were trained only with annotations in the relevant aggregation, and evaluated on the corresponding annotations (see Additional file 6 for the complete data set). For ABNER in particular (Table 14), we see that some combinations of categories seem to perform particularly badly, indicating that those categories may be particularly difficult to recognize. LingPipe (Table 15) has somewhat more consistent results across the various category groupings, through there is still significant variability. A possible explanation for lower performance with the polystar mappings may be that there is insufficient training data in those aggregations to derive a good model. All system performances were statistically different than the others, (p < 0.01); see Methods Section Statistics used for NLP tools performance differentiation for the details of the test performed.

**Table 14:** Precision/Recall/F1-score results for gene mention over CRAFT development set: ABNER with model retrained from the CRAFT public release data set

	**ABNER CRAFT** **star-STAR**			**ABNER CRAFT** **gene-GENE**			**ABNER CRAFT** **genestar-GENESTAR**			**ABNER CRAFT** **poly-POLY**			**ABNER CRAFT** **polystar-POLYSTAR**		
	**Prec**	**Recall**	**F1**	**Prec**	**Recall**	**F1**	**Prec**	**Recall**	**F1**	**Prec**	**Recall**	**F1**	**Prec**	**Recall**	**F1**
overlap	0.72	0.40	0.51	0.86	0.33	0.48	0.78	0.40	0.53	0.56	0.04	0.07	0.64	0.06	0.11
shared	0.72	0.40	0.51	0.86	0.33	0.48	0.78	0.40	0.53	0.56	0.04	0.07	0.64	0.06	0.11
subspan	0.72	0.40	0.51	0.86	0.33	0.48	0.78	0.40	0.53	0.56	0.04	0.07	0.64	0.06	0.11
strict	0.63	0.35	0.45	0.83	0.31	0.46	0.73	0.38	0.50	0.50	0.03	0.06	0.63	0.06	0.11

**Table 15:** Precision/Recall/F1-score results for gene mention over CRAFT development set: LingPipe with model retrained from the CRAFT public release data set

	**LingPipe CRAFT** **star-STAR**			**LingPipe CRAFT** **gene-GENE**			**LingPipe** **genestar-GENESTAR**			**LingPipe CRAFT** **poly-POLY**			**LingPipe** **polystar-POLYSTAR**		
	**Prec**	**Recall**	**F1**	**Prec**	**Recall**	**F1**	**Prec**	**Recall**	**F1**	**Prec**	**Recall**	**F1**	**Prec**	**Recall**	**F1**
strict	0.60	0.64	0.62	0.50	0.73	0.59	0.49	0.75	0.59	0.21	0.34	0.26	0.23	0.34	0.27
subspan	0.62	0.67	0.64	0.52	0.77	0.62	0.52	0.79	0.62	0.21	0.34	0.26	0.23	0.35	0.28
shared	0.62	0.67	0.64	0.52	0.77	0.62	0.52	0.79	0.62	0.21	0.34	0.26	0.23	0.35	0.28
overlap	0.62	0.67	0.64	0.52	0.77	0.62	0.52	0.79	0.62	0.21	0.34	0.26	0.23	0.35	0.28

For both systems, the best results are obtained when all of the various semantic classes are grouped together both for training and for evaluation, suggesting that the systems have done a reasonable job of generalizing over the different types of sequence mentions. When compared with the performance with distributed models, the LingPipe system performed better upon re-training, achieving a highest F-score of 0.64 as compared to 0.46 on the development set with the distributed models (Table 11). In contrast, the ABNER system had an overall drop in performance on retraining; it was able to achieve much better Precision at a substantial cost to Recall.

The LingPipe results after retraining are encouraging, and do slightly outperform the best out-of-the-box results we achieved with BANNER. We believe the modest improvements upon retraining may be due to how we structured the learning problem: due to overlaps among the different aggregation sets, we removed any existing annotations not in a given aggregation set before training. This means that the system cannot take advantage of constraints among different annotation types to improve the model for the target category. It is well-established in the machine learning community that learning multiple categories simultaneously generally results in better overall performance of the model. We look forward to more experimentation with learning NER models over the CRAFT data to better understand this behavior.

### Syntactic pre-processing: Sentence boundary detection, tokenization, and part of speech tagging

A number of steps in any text mining pipeline or machine learning algorithm are dependent on the accuracy of lower-level task performance. For this reason, we evaluated the performance of systems for sentence boundary detection, tokenization, and part of speech (POS) tagging.

The input to the sentence detectors was the original plain text (UTF-8 encoded) articles with no markup. The sentence-detected output was the input to the tokenizers. Each tokenizer was paired with its own sentence detection tool (i.e. the OpenNLP tokenizer uses OpenNLP sentence annotation, the LingPipe tokenizer uses LingPipe sentence annotation, etc.). Similarly, the input to the POS-taggers was sentence and token annotated data from the corresponding tools. All token, sentence, and POS annotations from the various tools were evaluated using the strict span matching criteria (see Table 13).

Sentence boundary detection was evaluated on the basis of precision/recall of character-based sentence boundary placement. Post-processing was performed that removed whitespace from character span counts at the end of sentence annotations and that removed empty-span sentence annotations. Table 16 shows the results for sentence boundary detection. The permutation test (see Methods Section Statistics used for NLP tools performance differentiation) showed that the difference in performance between LingPipe and the other two tools was significant (p < 0.01); the difference between OpenNLP and UIMA was not. The major difference between the high performance of LingPipe and the lower performances of OpenNLP and UIMA is that the former is able distinguish section headings from the surrounding text.

**Table 16:** Sentence boundary detection results on the CRAFT public release data (70% set)

**Sentence boundary detector**	**Precision**	**Recall**	**F-measure**
LingPipe	0.98	0.98	0.98
OpenNLP	0.87	0.74	0.80
UIMA-native	0.85	0.75	0.80

Tokenization and POS tagging were evaluated likewise. Table 17 shows the results for tokenization. The permutation test showed the performance of each tool to be significantly different than the others (p < 0.01). Here, we see that the default UIMA tokenizer actually outperforms the more specifically biomedical tokenizer of the PennBio framework; this likely stems from the treatment of punctuation in our annotation guidelines.

**Table 17:** Tokenization results on the CRAFT public release data (70% set)

**Tokenizer**	**Precision**	**Recall**	**F-measure**
UCompare OpenNLP	0.95	0.86	0.90
UIMA-native	0.96	0.93	0.95
PennBio	0.92	0.91	0.91
Offset Tokenizer	0.97	0.80	0.88

The results for POS tagging are in Table 18. The permutation tests showed that all system performances were significantly different (p < 0.01), except for LingPipe with the Genia model against OpenNLP. Here we see surprisingly poor performance, with none of the systems reaching even 0.8 F-score on the CRAFT data, well below state of the art for general English POS tagging. The highest-performing system (LingPipe with the Genia model) is a model specifically trained on biomedical text, indicating the importance of domain-relevant training material. The lowest performing models, LingPipe with the Brown model and with the MedPost model, have different tag sets, which greatly impairs their apparent performance when compared against the Penn Tagset used in CRAFT. To adjust for the different tagsets and provide an upper-bound notion of POS tagger performance, those tags that did not align with the gold standard set were removed from the evaluation for all four tools. These adjusted values are presented in parentheses in Table 18. Note that even OpenNLP and LingPipe with the Genia model have a higher upper-bound than their actual performance; this is because each have a small set of tags that do not align to the gold-standard tagset. The overall low performance even with those tools using the Penn Tagset, (i.e. UIMA and LingPipe Genia Model), is of concern for BioNLP systems, since much downstream processing makes use of POS-tagged data (note that in our parsing experiments below, we provide gold standard POS tags as input to the parsers whenever possible to avoid cascading errors).

**Table 18:** Part of speech tagging results on the CRAFT public release data (70% set)

**POS Tagger**	**Precision**	**Recall**	**F-measure**
LingPipe (Brown model)	0.59 (0.90)	0.58 (0.84)	0.59 (0.87)
LingPipe (MedPost model)	0.47 (0.88)	0.46 (0.83)	0.46 (0.85)
LingPipe (Genia model)	0.79 (0.88)	0.76 (0.85)	0.77 (0.87)
OpenNLP	0.82 (0.86)	0.74 (0.77)	0.78 (0.81)

Numbers in parentheses indicate the upper-bound performance potential of the tools, calculated by removing occurrences of tags that did not align to the gold-standard tagset.

### Parsing

We compared parsers under a variety of conditions related to (a) type of model and (b) type of output. We differentiated between parsers distributed with models built on non-biomedical text and parsers with models built on biomedical text. We differentiated between dependency parsers and constituency parsers. For parsers that could be trained, we retrained them on the CRAFT data (see Methods Section Data partitioning).

#### Constituency Parsing

Constituency parsers vary in their required input formats and allowable configuration. The required input format for each parser, which varied from one token/POS-tag pair per line to one sentence per line with specific delimiters between tokens and POS-tags, was extracted from the gold-standard treebanked parses from the public release CRAFT set. Parsers that could be configured to accept sentence-split, pre-tokenized, POS-tagged input were provided this pre-processed input derived from the gold standard. Parsers that could not were provided just the gold standard sentence-split input (Charniak-Lease and Charniak-Johnson parsers). The Charniak-Lease and Charniak-Johnson parsers are very similar. We show results from the older Charniak-Lease version of the parser because it was distributed with a model that was trained on biomedical text. The Stanford Parser accommodated a character encoding configuration and that was set to handle the input as UTF-8.

To evaluate full syntactic parses, we used the version of evalb provided with the Stanford Parser Java 1.6.6 package [45]. The evalb scoring categories are labeled bracket precision, (LB-P, number of correctly labeled and spanned constituents divided by number of constituents in parsed input), labeled bracket recall (LB-R, number of correctly labeled and spanned constituents divided by number of constituents in gold standard input), and F-score (LB-F), applied to each sentence as a whole. The values presented here are the sentence scores averaged over the section of corpus being tested. Except where noted, all comparisons of tools on the same dataset were statistically different (p < 0.01) using the permutation test (see Methods Section Statistics used for NLP tools performance differentiation).

Each parser struggled with a different small set of sentences that it could not parse, and the parse output of these sentences varied per parser. In some cases the parser output had to be manipulated manually to conform to a format that evalb could handle. Evalb skips any sentence for which the token count between the gold-standard sentence and the automatically parsed sentence does not match, and sentences that could not be parsed fall into this category. Additionally, some parsers retokenized input containing punctuation despite being given gold-standard tokenization and POS information; in some cases these alterations changed the token count, leading to higher counts of sentences that were not evaluated by evalb; this figure is shown in the ‘unevaluated count’ column of the tables.

Parsing results for parsers distributed with general English (non-biomedical) parsing models appear in Tables 19 and 20. The Mogura, Charniak-Lease and Charniak-Johnson parsers are distributed with models trained on biomedical annotated text (the biomedical model with the Charniak-Johnson parser was created by David McClosky [46]). Also, the Stanford 1.6.6 parser is released with a default model that includes training from sections of the GENIA corpus in addition to general English text [22]. The results from evaluation of the development set using these biomedical models are presented in Table 21. Comparing Tables 20 and 21, we see that, perhaps counter-intuitively, on the CRAFT development set the general English models outperform the biomedical models, even when the same underlying system is used. The exception is the Mogura parser, which had nearly identical performance in both cases. The results of the parsers using the biomedical models on the release set appear in Table 22. Note that we were unable to obtain successful parses on CRAFT with the Enju parser using the distributed biomedical model and so no results for that parser/model combination are included here.

**Table 19:** Results of constituent parsers using their distributed non-biomedical models on the CRAFT release set; labeled bracket precision (LB-P), recall (LB-R) and F-score (LB-F)

**Parser**	**LB-P**	**LB-R**	**LB-F**	**unevaluated count**
Berkeley	58.35	61.05	59.67	24
Bikel	63.34	65.27	64.29	5
Charniak-Johnson	56.97	49.92	53.21	166
Enju	57.76	59.87	58.80	612
Mogura	47.45	55.65	51.22	105
Stanford 1.6	57.70	62.31	59.92	4

**Table 20:** Results of constituent parsers using their distributed non-biomedical models on the CRAFT development set; labeled bracket precision (LB-P), recall (LB-R) and F-score (LB-F)

**Parser**	**LB-P**	**LB-R**	**LB-F**	**unevaluated count**
Berkeley	61.60	64.50	63.02	4
Bikel	63.97	65.82	64.89	2
Charniak-Johnson	62.51	65.55	64.00	59
Enju	71.93	43.56	54.26	8
Mogura	54.74	43.25	48.32	8
Stanford 1.6	60.76	64.70	62.67	3

**Table 21:** Results of constituent parsers using their distributed biomedical models on the CRAFT development set; labeled bracket precision (LB-P), recall (LB-R) and F-score (LB-F)

**Parser**	**LB-P**	**LB-R**	**LB-F**	**unevaluated count**
Charniak-Johnson	56.08	61.10	58.48	0
Charniak-Lease	55.53	59.77	57.57	2
Mogura	54.21	44.09	48.63	8
Stanford 1.6.6	61.10	62.65	61.87	2

**Table 22:** Results of constituent parsers using their distributed biomedical models on the CRAFT release set; labeled bracket precision (LB-P), recall (LB-R) and F-score (LB-F)

**Parser**	**LB-P**	**LB-R**	**LB-F**	**unevaluated count**
Charniak-Johnson	51.23	55.99	53.50	0
Charniak-Lease	53.28	57.43	55.28	8
Mogura	47.55	56.27	51.54	105
Stanford 1.6.6	59.49	61.81	60.63	10

For parsers that allowed retraining, we performed 5-fold cross-validation on the training set and report the performance on the development set; see Table 23. Not surprisingly, the parser performance using the CRAFT-retrained models showed a large improvement over those using the distributed models. The Berkeley parser showed greater improvement than the Stanford or Bikel parsers, with the best results of about 83% LB-F.

**Table 23:** Results of constituent parsers using retrained CRAFT models for each CRAFT fold and the development set compared to untrained results on the development set; labeled bracket precision (LB-P), recall (LB-R) and F-score (LB-F)

**Parser**	**Fold 0**	**Fold 1**	**Fold 2**	**Fold 3**	**Fold 4**	**Training Average**	**Dev Set**	**Dev Set Untrained**
**Berkeley**								
LB-P	82.75	92.02	84.63	83.70	83.85	85.39	83.98	61.60
LB-R	82.64	90.82	84.01	83.29	82.88	84.73	83.20	64.50
LB-F	82.70	91.41	84.32	83.49	83.36	85.06	83.59	63.02
**Bikel**								
LB-P	80.49	81.10	81.18	80.77	91.43	82.99	80.86	63.97
LB-R	79.68	79.77	80.10	80.46	91.06	82.21	80.44	65.82
LB-F	80.08	80.43	80.64	80.62	91.24	82.60	80.65	64.89
**Stanford 1.6.6**								
LB-P	75.65	75.86	77.71	76.21	77.86	76.65	76.17	60.76
LB-R	76.81	76.84	78.65	77.24	77.85	77.48	75.92	64.70
LB-F	76.23	76.34	78.18	76.72	77.86	77.07	76.04	62.67

#### Dependency parsing

While the CRAFT corpus has been syntactically annotated with constituent trees, the use of dependency parses rather than constituent parses is becoming increasingly common in biomedical natural language processing. Clegg and Shepherd [5] have argued that measuring parser performance through constituent-based accuracy fails to adequately distinguish between real differences in meaning derived from incorrect syntactic analysis and minor differences of convention that do not truly affect the output of text mining systems. Hence, we perform an analysis of constituency parses that have been translated to dependency structures. This also enables comparison of the CRAFT trees with the output of the dependency parsers. To do this comparison, the gold standard constituency parse was translated to a dependency representation.

Two kinds of dependency parses are evaluated here: parses that originated from a dependency parser and parses that originated from a constituency parser and were converted to dependency representations. Performing constituent-based parsing followed by conversion of the outputs to dependency trees has been shown to give higher accuracy than performing parsing directly to dependency trees for Stanford dependencies [47]. This is mostly because the dependency structures we are evaluating against are themselves converted from constituent-based trees. On the other hand, performing constituent-based parsing and doing the conversion is literally 100 times slower than performing dependency parsing directly (the Berkeley constituent-based parser takes 0.3 seconds per sentence; the ClearParser takes 2.5 milliseconds per sentence).

Like the constituent parsing, dependency parsers were provided gold standard tokenization and POS-tags extracted from the gold standard public release set of CRAFT. The output was evaluated using the standard measurements typically used at CoNLL for dependency parse evaluation. The labeled attachment score (LAS) corresponds to a complete comparison of the dependency structures in the system to the structures in the goal, for each sentence, requiring that individual tokens are assigned to the correct head, with the correct dependency relation. The unlabeled attachment score (UAS) relaxes the requirement that the dependency relation matches, only requiring association with the correct head. The labeled accuracy score (LS) requires that the dependency relations match, but relaxes the requirement of being assigned to the correct head.

Micro accuracy of a fold is calculated as in Equation 1, i.e. the accuracy across all individual gold standard dependencies in the fold. Macro accuracy is calculated as the average of accuracies across all *trees* in the relevant fold. We have not calculated accuracy averaged across individual documents, due to the differences in the number of sentences in the documents.

(1) $$\begin{array}{cc} \text{microaccuracy}= & \left(\text{\# of correctly classified dependencies}\right)\\ ÷(\text{total \# of dependencies}) \end{array}$$

For the dependency parser output, we report the individual score on each training fold, the average across the training folds, the score on the development set data for a model trained on the complete CRAFT training set, and the score on the development set data for the standard model for each parser trained on the Penn Treebank Wall Street Journal corpus (sections 2-21). Tables 24 and 25 show the results for the dependency parsers we tested.

**Table 24:** Micro-averaged results for dependency parsers on the CRAFT folds and dev set compared to untrained results on dev set; labeled attachment score (LAS), unlabeled attachment score (UAS), labeled accuracy score (LS)

**Parser**	**Fold 0**	**Fold 1**	**Fold 2**	**Fold 3**	**Fold 4**	**Training Average**	**Dev Set**	**Dev – WSJ model**
**MaltParser**								
LAS	85.81	86.29	87.08	86.13	86.26	86.34	86.04	69.78
UAS	87.94	88.43	89.16	88.18	88.16	88.39	87.91	73.42
LS	92.19	92.74	93.12	92.78	92.80	92.75	92.75	82.01
**MSTParser**								
LAS	85.65	86.37	86.89	86.08	86.29	86.28	86.70	**71.51**
UAS	87.96	88.57	89.04	88.21	88.43	88.46	88.86	75.08
LS	92.09	92.95	93.24	92.91	92.92	92.86	93.37	83.26
**ClearParser**								
LAS	86.46	86.99	87.94	87.12	87.23	**87.18**	**87.56**	70.43
UAS	88.23	88.81	89.62	88.82	88.86	88.89	89.11	73.62
LS	92.71	93.33	93.93	93.47	93.66	93.45	93.99	83.09

**Table 25:** Macro-averaged results for dependency parsers on the CRAFT folds and dev set compared to untrained results on dev set; labeled attachment score (LAS), unlabeled attachment score (UAS), labeled accuracy score (LS)

**Parser**	**Fold 0**	**Fold 1**	**Fold 2**	**Fold 3**	**Fold 4**	**Training Average**	**Dev Set**	**Dev – WSJ model**
**MaltParser**								
LAS	88.45	88.70	89.62	89.12	88.85	88.97	88.93	72.40
UAS	90.33	90.63	91.50	90.94	90.51	90.80	90.72	75.90
LS	93.43	93.78	94.23	94.16	93.93	93.92	94.03	82.73
**MSTParser**								
LAS	88.30	88.85	89.58	89.12	88.90	88.98	89.36	**75.99**
UAS	90.37	90.83	91.50	91.04	90.82	90.93	91.31	79.42
LS	93.32	94.06	94.37	94.25	93.98	94.03	94.52	85.73
**ClearParser**								
LAS	89.09	89.43	90.33	89.86	89.59	**89.68**	**90.09**	74.56
UAS	90.66	91.09	91.81	91.42	91.08	91.23	91.63	77.78
LS	93.89	94.37	94.88	94.65	94.57	94.50	94.99	85.17

Table 26 shows the results on the development set for the constituency parsers mapped to a dependency representation, evaluated with the same method as the strict dependency parsers, for comparison. These results were not as good as the strict dependency parse results, which we did not expect, based on [47]. However, since the constituent parsers we tested did not produce the function tags (e.g. -TMP, -LOC) that our system used for reliable constituent-to-dependency conversion of the CRAFT trees, they lost accuracy in the conversion step, particularly in getting the dependency labels correct. Thus, the labeled attachment scores of these two approaches are not directly comparable. We can more meaningfully compare unlabeled attachment scores, meaning we evaluate only on edges regardless of labels. We see that the Berkeley Parser results for UAS in Table 26 nearly approach the UAS results of the strict dependency parsers.

**Table 26:** Parsing accuracy of constituency parsers, evaluated on their generated dependency correspondences

**Parser**	**Dev Set**	**Dev – WSJ model**
**Berkeley Parser**		
(Micro) LAS	76.97	60.21
(Micro) UAS	88.11	70.66
(Micro) LS	83.13	72.68
(Macro) LAS	80.34	65.19
(Macro) UAS	91.04	75.57
(Macro) LS	84.98	75.63
**Stanford Parser**		
(Micro) LAS	72.13	58.42
(Micro) UAS	83.22	68.83
(Micro) LS	80.12	72.12
(Macro) LAS	75.87	62.10
(Macro) UAS	86.57	71.98
(Macro) LS	82.40	73.85

## Conclusions

We began this work by introducing two use cases for the CRAFT corpus, (a) evaluation of existing tools and (b) retraining of those tools. Our investigations have led to several conclusions.

### Algorithms and models differ in their generalizability

It is not controversial to state that different algorithms differ in their ability to train models that generalize to novel corpora. However, as the work of Banko and Brill [48] has shown, these differences may become apparent only as increasing amounts of data become available. We suspect that it is also the case that these differences may become apparent only as increasing numbers of genres become available. Prior work has looked at differences in performance based on training on the WSJ versus biomedical abstracts; the work reported here adds a new dimension to genre variability by introducing the full text of biomedical articles, which differ with respect to structure and content from both WSJ articles and biomedical abstracts [4].

### Tool performance is increased

As was shown in the sections on parsing, tool performance is increased when applications are re-trained on the data in the CRAFT corpus. This means that the bottleneck in performance that the field previously faced when trying to move from processing abstracts to processing full text can be overcome.

Our current results for retraining the gene mention recognition systems unfortunately did not show much improvement. We anticipate that these will improve significantly after some reconfiguration of the learning problem posed to the gene mention recognition systems, as described at the end of Section Gene mention recognition.

### CRAFT is a high quality resource

The work reported here has demonstrated that the data in the CRAFT corpus can be used to train high-performing models for a variety of language processing tasks. In addition, we have shown that there is high inter-annotator agreement for the syntactic annotation of the corpus (Table 1). Taken together, these results support the conclusion that the CRAFT corpus is itself of high quality.

### Building a state-of-the-art BioNLP system

Based on the experiments described here, there are several tools that stand out for consideration for inclusion within a BioNLP system targeted at full text biomedical publications. For sentence boundary detection, the LingPipe sentence boundary detector out-performed others by a significant margin. For tokenization, the default tokenizer within UIMA does a good job. None of the part-of-speech taggers did a great job without retraining, though the OpenNLP tagger had the highest precision. Given that gold standard POS tags were provided to the parsers in most cases, it could be expected that use of a low-performing tagger would result in lower than reported parsing accuracy in a natural setting. However, the ClearParser dependency parser with CRAFT-trained model would be an excellent choice; the Berkeley parser with CRAFT-trained model should work well for constituency parsing of full text. Finally, for gene mention recognition BANNER appears to provide good out-of-the box performance, while LingPipe responded well to re-training. We hope to retrain BANNER on CRAFT in the near future to see additional performance gains.

### The effect of differing annotation guidelines

A possible reason for the differing performance of various tools on this full-text corpus is differences in annotation guidelines. However, this can be ruled out as the explanation for all differences. In previous work, we showed that performance differences, and sometimes quite drastic ones, manifest themselves when tools are evaluated separately on paper abstracts and paper bodies [4]. Since the annotation guidelines were identical for all parts of the articles, these differences cannot be due to differences in annotation guidelines—the only variable in this study was abstracts versus article bodies. We also note that although differences in tag sets could explain some of the differences in performance of part of speech taggers when applied to our full-text corpus, it clearly cannot explain all of it, since performance differences were noted even when the tag sets were the same.

### The future of BioNLP with the availability of CRAFT

We retrained a relatively small set of tools for this study (even if a larger set than in previous studies); it is exciting to think what advances could be made if additional tools are retrained on this corpus, and if different strategies are explored for taking advantage of the annotations. Furthermore, we look forward to still more annotation of this material by us and by other groups to support richer models integrating different aspects of language, including discourse and pragmatics.

### Ongoing and future work

We are currently producing a number of additional sets of annotations for the CRAFT corpus: 

Relations: Assertions of relationships between semantic types already annotated in the corpus are in progress.

Coreference: All coreference in the corpus is being annotated. The process and guidelines are discussed in [49].

Discourse: Discourse functions have been marked at the sentence level.

Parentheses: All parenthesized text is being classified according to an ontology of parenthesis contents in scientific journal articles. The ontology and preliminary scores for a classifier for the ontology concepts are described in [50].

Evidence sentences: All sentences used as evidence for GO annotations at MGI are being marked.

These new sets of annotations will be released as they are completed.

## Methods

### Data

We used a pre-0.9 release of the CRAFT data set. CRAFT is comprised of 97 full-text journal articles selected from the PubMed Central Open Access subset [51] for their relevance to the Mouse Genome Informatics database. The articles were selected by retrieving all articles that (1) were used as evidence for at least one Gene Ontology annotation by MGI, and (2) were available Open Access. This intersection yielded 98 full-text journal articles. One of the articles was only available as a scanned PDF, leaving 97 articles that could be subjected to natural language processing. For details about the concept (semantic) annotation of the CRAFT corpus, please see [14]. We have previously argued [6] that the open access literature, and specifically the CRAFT corpus, is representative of the general biomedical literature, hence the experiments reported here should generalize to comparable biomedical corpora.

### Data partitioning

We divided the CRAFT corpus into three partitions, one containing 70% of the data and two each containing 15% of the data. We used the 70% partition for the experiments reported here. We refer to this set as the “public release” CRAFT set. The two partitions containing 15% of the data have been held back for use in shared tasks. The data sets were randomly generated, but we confirmed that they are balanced such that similar ratios of semantic classes of named entities occurred in each split. One-way ANOVA statistics were calculated for each ontology annotated in CRAFT. Based on these tests, annotation counts in the three random subsets were not statistically different from the complete set.

To support retraining, we further subdivided the experiment partition into 6 folds: 5 folds of 12 files each comprising a training set, and a development set of the remaining 7 files. The folds were selected in simple ID order. Table 27 shows the distribution of the data across the folds. Additional file 7 provides lists of the document IDs corresponding to each fold and the development set.

**Table 27:** Distribution of data across the folds

**Fold**	**Number of Sentences**	**IDs of files in fold**
Fold 0	3,066	11532192 - 15005800
Fold 1	3,990	15040800 - 15630473
Fold 2	3,951	15676071 - 16110338
Fold 3	3,723	16121255 - 16507151
Fold 4	4,200	16539743 - 17083276
Training	18,930	11532192 - 17083276
Development	2,780	17194222 - 17696610

Five-fold cross validation was performed using these folds for all tools that support retraining. To avoid systems having to learn all of the fine-grained distinctions of the Sequence Ontology captured in CRAFT, we performed aggregation of the semantic classes prior to the re-training. In other words, the “star-STAR” results reflect a model in which all semantic categories in CRAFT are aggregated into a single class that is learned by the system. We then compare to the corresponding aggregation set in the evaluation. After consistency of the results from five-fold training was confirmed, a final model was trained on all five folds. Evaluation was then performed on the development set.

### Inter-annotator agreement

All inter-annotator agreement (IAA) statistics for syntactic annotation were calculated with the original version of the evalb bracket scoring program using a modified version of the Collins parameter file [52], which matches constituent (bracket) placement and node labels, disregarding function tags, punctuation, symbols, empty categories and indexation. IAA statistics reported are defined as $\text{precision}(A1)=\left|\left\{A1\}\cap \{A2\right\}\right|/\left|\left\{A1\right\}\right|$ and $\text{recall}(A1)=\left|\left\{A1\}\cap \{A2\right\}\right|/\left|\left\{A2\right\}\right|$, where {*A*1} and {*A*2} are annotation sets; these were calculated for the annotation sets of six fully treebanked files marked up by the three senior annotators (two files per pairing) in the configurations {*A*1}−{*A*2},{*A*1}−{*A*3} and {*A*2}−{*A*3}. Additionally, IAA was calculated between annotators and the gold standard (which was maintained by the lead syntactic annotator) and also between automatically created OpenNLP annotations and the gold standard.

### NER Tools

The NER tools tested here were run in a UIMA 2.2.2 environment using locally developed adapting software: BioNLP-UIMA 1.4 (not yet released publicly) (http://bionlp.sourceforge.net). Pre-syntactic annotations of tokens and sentences were provided as input from the CRAFT Treebank gold standard files. The UIMA pipelines were defined in Java using uimaFIT 1.0 [53]. This allowed us to more easily run all the variations and collate the output. The Java version used was 1.6.0. The gold standard data was read with libraries from Knowtator 1.7.4 [54], and Protege 3.3.1 [55]. We used ABNER version 1.5 [56], the version of BANNER [57] available in the repository as of October 25, 2011, and LingPipe 3.9.3 [58]. ABNER and LingPipe training was accomplished using software developed in-house to produce the input files to training code provided with the respective NER tools. The systems were trained on the 60 files in the training set, and tested on the development set, as described in Section Data partitioning.

### Syntactic pre-processing tools

These lower-level task experiments were run within the UIMA environment, and all annotations were brought into alignment with a common UIMA type system defined in-house [59]. The tools used in this work are the OpenNLP sentence-detector, tokenizer and POS-tagger v 1.3.0 [60], the LingPipe sentence-detector and POS-tagger v 3.9.3 [58], the default versions of the UIMA-native sentence-detector and tokenizer shipped with UIMA v 2.2.2 [61], the PennBio tokenizer v 0.5 [62], and the Offset tokenizer that is distributed with ConceptMapper v August 2008 [63].

### Parsers

The constituent parsers tested in this work are the Berkeley Parser v 1.1 [64], the Bikel Parser v 1.0 [65], the Charniak-Lease Parser release date July, 2005 [66], the Enju and Mogura Parsers v 2.4.1 [67], the McClosky-Charniak Parser v a8fca3a4d59b [68], and the Stanford Parser v 1.6 and 1.6.6 [45]. They are evaluated using the version of evalb provided with the Stanford Parser Java 1.6.6 package [45].

The dependency parsers tested in this work are the MaltParser v 1.5.2 [69], the MSTParser v 0.4.3c [70], and the ClearParser v 0.3 [71]. They are evaluated using the evaluation script available as part of the ClearParser package, (http://code.google.com/p/clearparser/,classDepEvaluate) [72].

The tool used to translate constituent parses to dependency parses was the Clear Constituent-to-Dependency Converter (http://code.google.com/p/clearparser/,classPennToDep) [72]. The conversion tool was provided with some conversion rules specific to the CRAFT treebank representation where it varies from the original Penn Treebank representation. For instance, the CRAFT representation of formulas, e.g.:

(S-FRM

(NP-SBJ (NN n))

(VP (SYM =)

(NP (CD 7))))

was converted to a dependency relation of the form:

‘‘='' -SBJ-> ‘‘n''

-OBJ-> ‘‘7''

The headrules required to achieve the appropriate conversion are also included with the CRAFT release. The conversion scheme is inherited from the LTH tool used for the CoNLL 2007-9 [73].

### Statistics used for NLP tools performance differentiation

Differences in system performances for the NER tools, the pre-syntactic processing tools, and the parsing tools were statistically verified using a permutation test to test the difference in F-scores between two tools. For NER and pre-syntactic processing tools, the number of permutations was 10,000. Due to excessive processing time, the parser performances were based on 1000 permutations instead. All significance claims are based on p < 0.01.

## Availability

The corpus has been made available at http://bionlp-corpora.sourceforge.net/CRAFT/index.shtml.

## Abbreviations

CRAFT: Colorado richly annotated full text; NLP: Natural language processing; PTB: Penn treebank; UIMA: Unstructured information management architecture; POS: Part-of-speech; IAA: Inter-annotator agreement; LAS: Labeled attachment score; UAS: Unlabeled attachment score; LS: Labeled accuracy score; P: Precision; R: Recall; F: F(1) score; LB-P: Labeled bracket precision; LB-R: Labeled bracket recall; LB-F: Labeled bracket f-score.

## Competing interests

The authors declare that they have no competing interests.

## Author’s contributions

AL, CW, ME, NX and MP planned or participated in the syntactic annotation of the corpus. AL and CW wrote the first versions of sections of the manuscript pertaining to syntactic annotation. KV planned and directed the NLP experiments, performed data analysis, and wrote or integrated text for the sections pertaining to NLP experiments. KV, HLJ, CR, JDC, and CF participated in the experiments and analysis of NLP tools. WAB participated in the corpus data processing. YM performed statistical analysis of partitions. MB planned and participated in the gene annotation. KBC was involved with planning the syntactic annotation and related experiments, and contributed to the writing of the manuscript. LEH supervised all aspects of the work. All authors read and approved the final manuscript.

## Supplementary Material

Additional file 1 Full tagset used in the CRAFT corpus. Click here for file

Additional file 2 CRAFT addendum to syntactic annotation guidelines. CRAFT addendum to PTB2 and PennBioIE syntactic annotation guidelines. Click here for file

Additional file 3 Morphosyntactic data type counts. Counts of each node, tag, and empty category in the CRAFT corpus. Click here for file

Additional file 4 Semantic class aggregations. The specific definitions of labeled aggregations of semantic categories used in evaluation. Click here for file

Additional file 5 Full gene mention results for distributed models. Full results for gene mention systems with their distributed models, for all semantic class mappings and system/model combinations. Click here for file

Additional file 6 Full gene mention results for retrained systems. Full results for gene mention systems with models retrained on the CRAFT data, with all semantic class mappings and system/model combinations. Click here for file

Additional file 7 Folds and development set. Lists of the document identifiers corresponding to each fold and to the development set. Click here for file
