# Supplementary material for: A corpus of full-text journal articles is a robust evaluation tool for revealing differences in performance of biomedical natural language processing tools
Source: BMC Bioinformatics. 2012 Aug 17;13:207. doi: 10.1186/1471-2105-13-207 (PMC3483229; doi:10.1186/1471-2105-13-207)
Supplement: Additional file 1 — Full tagset used in the CRAFT corpus. [file 1471-2105-13-207-S1.pdf]

CRAFT Syntactic Tag Set:

Syntactic Nodes:	S, NP, VP, PP, SBAR, SBARQ, SQ, SINV, NML, LST, PRN, PRT, QP, ADVP, ADJP, FRAG, WHNP, WHPP, WHADVP, WHADJP, CONJP, INTJ, NAC, RRC, UCP, X, EDITED, CIT, TITLE, HEADING, CAPTION
Empty Categories:	*, *PRO*, *T*, *RNR*, *ICH*, *EXP*, *U*, 0, *?*, *NOT*
Function Tags:	-SBJ, -TMP, -TPC, -PRP, -PRD, -CLR, -SEZ, -LOC, -DIR, -MNR, -ADV, -LGS, -NOM, -DTV, -VOC, -BNF, -EXT, -CLF, -HLN, -TTL, -IMP, -ETC, -UNF, -FRM

Syntactic Nodes:

S – sentence
NP – noun phrase
VP – verb phrase
PP – prepositional phrase
SBAR – subordinate or relative clause
SBARQ – *wh*- questions
SQ – question
SINV – subject-auxiliary inversion and some topicalizations
NML – nominal sub-constituents
LST – list marker
PRN – parenthetical
CIT - citation
PRT – particle
QP – quantifier phrase
ADVP – adverb phrase
ADJP – adjective phrase
FRAG – fragment.
WHNP – *wh*- noun phrase
WHPP – *wh*- prepositional phrase
WHADVP – *wh*- adverb phrase
WHADJP – *wh*- adjective phrase
CONJP – multi-word conjunction phrase
INTJ – interjection
NAC – not a constituent
RRC – reduced relative clause
UCP – unlike coordinated phrases
X – unknown or unbracketable
TITLE - journal title
HEADING - section heading
CAPTION - table or figure caption

Empty Categories:

* -- passive movement and subject raising
PRO -- subject and object control
T -- *wh*- movement, topicalization
RNR -- right node raising
ICH -- interpret constituent here
EXP -- pseudo-attach expletive
U -- unit
0 – null complementizer and null *wh*- operator

? -- elided material
NOT -- gapping anti-place holder

Function Tags:
-SBJ – surface subject
-TMP – temporal
-TPC – topicalized
-PRP – purpose
-PRD – predicate
-CLR – used with S to denote small clause or secondary predicate
-LOC – locative
-DIR – direction of action
-MNR – manner of action
-ADV – adverbial
-LGS – logical subject (of passive construction)
-NOM – nominal sentence
-DTV – dative
-VOC – vocative
-BNF – beneficiary
-EXT – existential “it”
-CLF – it-cleft
-TTL – title
-IMP – imperative
-ETC – etcetera
-FRM – formula

CRAFT Part of Speech Tag Set:

Part of Speech Tags:	VB, VBZ, VBP, VBD, VBN, VBG, RP, LRB, RRB, NN, NNS, NNP, NNPS, IN, DT, TO, CC, MD, JJ, JJR, JJS, RB, RBR, RBS, PRP, PRP$, LS, EX, PDT, SYM, CD, UH, POS, FW, WP, WP$, WRB, WDT, HYPH, AFX, XX, ., ,, :, ``, "

POS tags:
VB – verb, base form
VBZ – verb, 3rd person singular present tense
VBP – verb, non 3rd person singular present tense
VBD – verb, past tense
VBN -- verb, past participle
VBG – verb, present participle
RP – particle
-LRB- -- left (paired) bracket or braces
-RRB- -- right (paired) bracket or braces
NN – singular noun
NNS – plural noun
NNP – proper noun, singular

NNPS – proper noun, plural
IN – preposition or subordinating conjunction
DT – determiner
TO – infinitive “to”
CC – coordinating conjunction
MD – modal
JJ – adjective
JJR – comparative adjective
JJS – superlative adjective
RB – adverb
RBR – comparative adverb
RBS – superlative adverb
PRP – personal pronoun
PRP$ -- possessive pronoun
LS – list item
EX – existential “there”
PDT – predeterminer
SYM – symbol
CD – cardinal number (includes Roman numerals and spelled-out numbers)
. – sentence final punctuation
, -- comma
: -- colons and semicolons used to separate one statement from another and em-dashes
`` -- opening quote
'' – closing quote
UH – interjection (ex. “please”)
POS – possessive marker (‘s or ‘)
FW – foreign word
WP – *wh*- pronoun
WP$ -- *wh*- pronoun, possessive
WRB – *wh*- adverb
WDT – *wh*- determiner
HYPH – hyphen or slash used as punctuation
AFX – affixes
XX – undecipherable material
