# Supplementary material for: A corpus of full-text journal articles is a robust evaluation tool for revealing differences in performance of biomedical natural language processing tools
Source: BMC Bioinformatics. 2012 Aug 17;13:207. doi: 10.1186/1471-2105-13-207 (PMC3483229; doi:10.1186/1471-2105-13-207)
Supplement: Additional file 2 — CRAFT addendum to syntactic annotation guidelines. CRAFT addendum to PTB2 and PennBioIE syntactic annotation guidelines. [file 1471-2105-13-207-S2.pdf]

CRAFT addendum to PTB II and Penn BioIE guidelines

Nianwen Xue, Arrick Lanfranchi, Colin Warner, Amanda Howard, Tim O'Gorman

Section 1: Coordination

1.1 NP coordination as an alternative to *P*

P is used in the Penn BioIE project as a place-holder for a distributed premodifier or head, and it is used exclusively in coordinated nominal constructions. The motivation for this is to ensure that each entity in a sentence corresponds to a node in the parse tree. This has proven to be very difficult in practice and we've decided to look for an alternative structure that represents the same information but with a simpler structure. Here is an example where *P* is used in the Penn BioIE project:

```
(NP  (NP k –
          (NML-1 *P*))
      and
     (NP N –
          (NML-1 ras)))
```

We are proposing this structure as an alternative representation:

```
(NP (NML K -
             and
             N -)
     ras)
```

This structure would represent the fact that 'K-' and 'N –' are both modifiers of 'ras'. A consequence of this structure is that the entities 'K-ras' and 'N-ras' no longer correspond to a node in a parse tree. However, in a distributed interpretation where each conjunct in the coordination structure modifies the head individually, which is the correct interpretation here, a simple inference rule can be applied to derive the entity 'K-ras' and 'N-ras'. Our view is that it is too costly to enforce the rule that there should be a node for each entity using the place-holder *P* as it done in the Penn BioIE project. More generally, we believe that allowing well-understood mismatches between different layers of linguistic representation would simplify the representation for each layer.

In the Penn BioIE project, shared premodifiers that have a distributed interpretation also lead to the use of *P*, e.g.,

```
(NP (NP (ADJP-1 enhanced)
        CYP2C9 production)
    and
    (NP (ADJP-1 *P*)
        (NML 11,12 EET)
        production))
```

It becomes more complicated when there are multiple shared premodifiers, where a place-holder *P* has to be postulated for each premodifier:

```
(NP (NP (ADJP-1 cultured)
        (NML-2 rat)
         pancreatic acinar cells)
    and
    (NP (ADJP-1 *P*)
        (NML-2 *P*)
        hepatocytes))
```

When both the head and premodifier are coordinated structures, creating a node for each entity is simply impossible. For example, in “the N- and K- ras cells and tumors”, there are four entities “N-ras cells”, “K-ras cells”, “N-ras tumors” and “K-ras tumors”. The best effort from the Penn BioIE project yields this structure:

```
(NP the
    (NML (NML  (NML-1 (NML N-
                           (NML-2 *P*))
                      and
                     (NML K-
                          (NML-2 ras)))
               cells)
         and
         (NML (NML-1 *P*)
              tumors)))
```

Instead of explicitly representing all four entities, this structure only explicitly represents “N-ras and K-ras cells” and “N-ras and K-ras tumors”. In order to derive all four entities from the parse tree, it will have to resort to an inference mechanism like the rule we have outlined above. If this inference mechanism has to be applied anyway, it seems to make sense to apply it consistently in all cases and simplify the representation of the

coordinated nominal structures. Therefore we will represent the above three examples as follows:

```
(NP enhanced
      (NML (NML CYP2C9 production)
               and
              (NML (NML 11,12 EET production))

(NP cultured
      rat
      (NML (NML pancreative acinar cells)
               and
               (NML hepatocytes)))

(NP the
      (NML N – and K -)
      (NML cells
               and
               tumors))
```

1.2 A note about NML

NML is a label that is added in the Penn BioIE addendum to represent sub-NP structures. We will adopt this label in our annotation. Although there are rules about where NML should be used, the addendum does not explicitly state the distinction between NML and NP. This section attempts to clarify that distinction.

PTB allows flat NP structures when there is a strictly right-branching structure where each daughter of the NP forms a constituent with everything on its right. For example,

(NP primary liver cancer)

is an implicit representation of

```
(NP primary
      (NODE liver
                (NODE cander)))
```

The purpose of the flat structure is to improve readability when the structure is completely predictable. Any non-right-branching structures have to be explicitly represented with nodes labeled NML:

```
(NP (NML Cytochrome P450)
      isoenzyme)
```

```
(NP selective
      (NML seratonin reuptake)
      inhibitor)
```

So far so good. Everything about NML up to this point is clear. However, since NPs, like other linguistic structures, are recursive, a sub-NP structure can also be an NP itself. The question is when a constituent should be labeled NP and when it should be labeled NML. For example, when two NPs are coordinated to form a larger NP, NML is **NOT** used:

```
(NP (NP liver cells)
      and
      (NP hepatocytes))
```

However, when the coordinated nominal elements are subconstituents of a larger, single NP, NML is used instead:

```
(NP  (NML (NML Hong Kong)
             -
             (NML Palau))
       trade)
```

In CRAFT, as in PTB2, NP adjunction is represented by NP rather than NML when there a no nominal sub-constituents.

```
(NP (NP loss)
      (PP of
            (NP hybridization)))
```

```
(NP (NP the patient)
       (VP seen
           (NP * )
           (NP-TMP yesterday)))
```

When this larger NP is a premodifier, it is labeled NML:

```
(NP the
    (NML (NML guanine)
         (PP to
             (NP cytosine)))
    transformation)
```

It is important to note that NML is not a “full” NP, but rather a piece of an NP. For instance, NML can never contain a determiner. This distinction is tricky because, particularly with proper name NMLs, material marked NML could also stand on its own as NP, but in the particular context in which we find them they are constituents that are really only part of an NP. For example, in the citation "(Anthony Nicholson, the Jackson Laboratory, personal communication)," "the Jackson Laboratory" is an NP on its own

(NP the Jackson Laboratory)

However, as a modifier, as in "The Jackson Laboratory foundation stock" it is annotated as a NML,

```
(NP the
    (NML Jackson Laboratory)
    foundation stock)
```

1.3 Adding other coordinated structures.

A separate and yet related issue when deciding on how to handle shared modifiers or head is the representation of coordination structures because coordination is the main mechanism for sharing modifiers or head. In the PTB II guidelines as well as the Penn BioIE addendum, certain coordination structures are left flat for legibility. For example, if the coordinated elements are single tokens that share some premodifiers, then the structure is left flat:

(NP combined washings and brushings)
(NP the dogs and cats)

(NP 11 dogs and cats)

In CRAFT, we explicitly show the scope of "the" (which is modifying both "cats" and "dogs"), by putting a NML node around "cats and dogs":

```
(NP the
    (NML cats and dogs))
```

In this way, we more closely align the annotation of these single-token coordinated heads with existing PTB2a policy regarding the use of NML in multi-token coordinated phrases with shared premodifiers:

```
(NP the
    (NML (NML grey cats)
         and
         (NML brown dogs)))

(NP the
    (NML (NML pupil)
         and
         (NML optic nerve)))
```

Leaving such coordination structures flat means less keystrokes or mouse clicks for annotators, but it often poses problems for users who are used to using the Treebank data as is without having to read the bulky guidelines to learn about the linguistic nuances. For example, when converting phrase structure to dependency structure, one would have to recognize the coordinate structure to identify the head.

This has implications for how to represent coordination structures for other categories as well, if we want to be consistent across all categories. The same argument from a user's perspective applies to coordinated VPs as well. Previously, in the PTB, shared adjuncts for coordinated VPs are left at the conjunction level:

(S (NP-SBJ-1 the company)
   (VP expects
        (S (NP-SBJ-1 **PRO***)
           (VP to
                (VP (VP obtain
                          (NP regulatory approval))
                     and

```
              (VP complete
                  (NP transaction))
              (PP by
                  (NP year-end)))))))
```

Notice that the PP "by year-end" is shared by both VPs "obtain regulatory approval" and "complete transaction", but is attached at the same level as those two VPs to form a flat structure. Like the flat coordinated nominal structures, this could also be a potential problem the user tries to convert this into a dependency structure. Our proposal is to add a layer of VP so that the PP modifier and the coordinated VP are at different levels of attachment. The added VP is in bold and underlined. This way, it will easier for the user to detect coordination structures and determine what the head is for such structures.

```
(S (NP-SBJ-1 the company)
   (VP expects
       (S (NP-SBJ-1 PRO*)
          (VP to
              (VP (VP (VP obtain
                          (NP regulatory approval))
                      and
                      (VP complete
                          (NP transaction)))
                  (PP by
                      (NP year-end)))))))
```

Shared modifiers can also occur at the S level. When two clauses share a modifier, the modifier is attached at the coordination level. It poses similar problems for phrase structure to dependency structures conversion:

```
(S (SBAR-ADV Although X)
   either
   (S Y)
   or
   (S Z))
```

In order to make this consistent with our treatment of coordinated NPs and VPs, we are also adapting this treatment from PTB.

```
(S (SBAR-ADV Although X)
   (S either
```

```
        (S Y)
        or
        (S Z)))
```

In some cases, the shared modifier is attached at the level of the first clause in the PTB even if it has scope over both coordinated clauses:
[This is no longer true for current PTB projects. Modifiers shared across sentences are now left loose at coordination level. I've attempted to correct the tree below, but these examples are now exactly parallel to the "Although X…" examples above, maybe we don't need both?]

```
(S (PP-TMP After
                  (NP (NP puncture)
                       (PP of
                          (NP (NP coagulated blood)
                               (PP from
                                   (NP the corpora cavernosa)))))
   (S (NP-SBJ-1 urine retention)
      (VP developed))
   and
   (S (NP-SBJ-1 a suprapubic catheter)
      (VP had
          (S (NP-SBJ-1 *)
             (VP to
                 (VP be
                      (VP introduced
                          (NP-1 *)
                          (ADVP temporarily)
                          (PP-PRP for
                                   (NP urine drainage)))))))))
```

Our proposal is to adjoin the temporal PP to the coordinated S structure as follows:

(S (**PP-TMP** After

```
                  (NP (NP puncture)
                       (PP of (NP (NP coagulated blood)
                                   (PP from
                                       (NP the corpora cavernosa))))))
   (S (S  (NP-SBJ-1 urine retention)
          (VP developed))
      and
      (S (NP-SBJ-1 a suprapubic catheter)
```

```
    (VP had
        (S (NP-SBJ-1 *)
            (VP to
                (VP be
                    (VP introduced
                        (NP-1 *)
                        (ADVP temporarily)
                        (PP-PRP for
                                (NP urine drainage))))))))))
```

The following is a summary of the changes and modifications in annotation style to explicitly annotate all coordination structures:

i). single-token coordinated heads inside NP with shared premodifier(s) ()

old style:

```
(NP the cats and dogs)
(NP k-ras cells and tumors)
(NP (NML n-ras and k-ras) cells and tumors)
```

new style:

(NP the (**NML** cats and dogs))
(NP k-ras
    (**NML** cells and tumors))
(NP (NML n-ras and k-ras)
     (**NML** cells and tumors))

old style same as new style:

```
(NP (NML k-ras and n-ras) cells)
(NP the (NML (NML magazines)
        and
        (NML heavy books)))
(NP the (NML (NML Penn undergraduates)
        and
        (NML Stanford postgraduates)))
(NP Bob, Joan, and Nancy)
(NP (NP Bob), (NP Joan), and (NP Mrs. Smith))
```

ii). Adjunction of shared adjuncts to coordinated VP and S

Shared adjunct before coordinated S:

```
(S (SBAR-ADV Although X)
   (S (S Y)
      and
     (S Z))
```

Shared adjunct after coordinated S:

```
(S (S (S X)
       and
      (S Y))
  (SBAR-ADV Z))
```

Shared adjunct after coordinated VP:

```
(S (NP-SBJ We)
   (VP (VP (VP ate
                 (NP food)
             and
            (VP drank
                 (NP drinks))
        (NP-TMP last night)))
```

Shared adjuncts before coordinated VP will remain the same:

```
(S (NP-TMP Yesterday)
  (NP-SBJ We)
  (ADVP rather quickly)
  (VP (VP ate
           (NP food))
       and
      (VP drank
           (NP drinks))))
```

Section 2: Headings, Titles, and Captions

In Penn Treebank II, sentence fragments are labeled FRAG at the top level. Since the data we are dealing with are journal articles and books, we are using more informative labels for things that would have been tagged FRAG based on PTP II guidelines. These labels are for things that are important in structuring a journal article or a book. CRAFT had created TITLE, HEADING, and CAPTION node labels to denote these sections of journal articles:

These new node labels expand upon the –HLN tag used in newswire treebanks. Fragments are still labeled FRAG in CRAFT as they would be in other treebanks, but get this additional node on top of that.]

2.1 Journal title: TITLE
((TITLE (NP PLoS Genetics)))

2.2 Section headings: HEADING
(HEADING (NP Abstract))

(HEADING (NP Introduction))

(HEADING (NP Results))

2.3. Figure, Table, and Picture Captions: CAPTION

```
(HEADING (NP Figures and Tables))
(HEADING (NP Figure 1))
(CAPTION
   (S (NP-SBJ The MouseSox1βgeo Allele)
      (VP Reveals
         (NP (NP the Requirement)
              (PP of (NP SOX1)))
         (PP in
            (NP (NP the Development
                  (PP of  (NP VS Neurons)))))))
```

These nodes require internal structure the same as other main text nodes, however, TITLE, HEADING, and CAPTION nodes have only one daughter. In cases where titles, headings or captions are not complete sentences FRAG may be used to make a single constituent of the daughter nodes.

(TITLE (FRAG (NP Complex Trait Analysis of the Mouse Striatum)

```
:
(S Independent QTLs Modulate Volume and Number)))
```

Section 3. Parentheticals, References and Citations

3.1 Adding primary label **CIT** for inline citations

In Penn BioIE Addendum (Section 7.2), citations are annotated as follows.:

(PRN ((FRAG Shelton et al., 1983)))

Since citations are pervasive in journal articles and books, we are adding a **CIT** tag for inline citations. The internal structures for citations are flat:

(CIT Shelton et al., 1983)

CIT applies only to author references that occur inside of parentheses. All other, non-parenthetical references are bracketed as normal text.

```
(VP reviewed
   (NP-1 *)
   (PP in (NP (NP (NP Furumura)
                 (ADVP-ETC et al.))
              (NP-TMP 1996))))
```

3.2 Expansion of PRN

We have expanded its use to include citations that consist only of page numbers. If the sentence contains only one parenthetical at the end of the sentence, then this is a daughter of the VP; otherwise, it is within whichever node it seems to be modifying.

```
(S      (NP-SBJ These mutations
        (VP shift
                (NP (NP the spectral profiles
                        (PP of
                                (NP the translation products))))
                (PRN [ (NP 4 , 11) ])
         .)
```

```
(S        (NP-SBJ-1 R1 ES cells
                   (PRN [(NP 20)]))
          (VP were
               (VP maintained
                    (NP-1 *)
                    (PP under
                        ( NP standard culture conditions ))
                    (PP in
                         (NP (NP the presence)
                              (PP of
                                   ( NP LIF)))))
           .)
```

In the case of two separate consecutive parentheticals, each gets a separate node:

```
(S (NP-SBJ Pierce)
    (VP has designed a set of ...
        (PRN (  (NP Fig 1.)  )
        (PRN (NP [ 25 ] )))
```

We are also using PRN for extra-syntactic material such as references to illustrations or company of origin.

```
(S        (NP-SBJ The embryo
          (VP is
                  (ADVP exclusively )
                  (ADJP-PRD ECFP+ )
                  (SBAR-ADV whereas
                            (S        (NP-SBJ its placenta
                                      (PRN [(PP-LOC to
                                                      (NP the left
                                                              (PP of
                                                                      (NP the embryo))))
                                              ] ))
                                      (VP is
                                              (ADVP predominantly)
                                              (ADJP-PRD EYFP+)))))
          .)
```

```
(S      (NP-SBJ Cells
        (VP were
                (ADVP clearly
                (ADJP-PRD distinguishable
                (PP within
                        (NP a mixed population
                (PRN [(NP Fig.
                        (NML 1a , 1b , 1c , 1d ) ]))
        .)
```

```
(S      (NP-SBJ-1 Images)
        (VP were
                (VP processed
                        (NP-1 *)
                        (S-ADV
                                (NP-SBJ *PRO*)
                                (VP using
                                        (NP Photoshop software
                                                ( PRN [(NP Adobe Systems )] )))))
        .)
```

If a PRN contains different elements, they are not joined with a parent node.

```
(S (NP-SBJ TMPred analysis)
   (VP predicts
       (NP (NP (NP a protein structure)
                (SBAR (WHNP-1 that)
                        (S (NP-SBJ-1 *T*)
                           (VP is
                               (ADJP nearly identical
                                       (PP to
                                           (NP MCOLN1)))))))
            ,
            (VP containing
                (NP (NP_6 transmembrane domains)
                     (PP with
                         (NP (NP the (NML N- and C ) -termini)
```

```
                                    (VP residing
                                         (PP-LOC in
                                                    (NP the cytoplasm)))))))))
(PRN -LRB-
      (NP Fig. 2)
      -RRB-)
(PRN -LRB-
      (NP 9)
      -RRB-)
 .)
```

Section 4. Miscellany

4.1 (Partial) Eliminating functionary tag **–CLR**

The functional tag **–CLR** is used to label prepositional phrases that can be interpreted as an argument for a verb. Whether a PP is an argument or not to a large extent depends on the specific verb and it is hard to make a general characterization about the nature of this category without referring to this verb. Therefore the Penn BioIE Addendum has a long list of verbs that take a PP that can be labeled **–CLR**. Below is an example of PP tagged **–CLR**:

```
(VP account
     (PP-CLR for
               (NP her disappearance)))
```

We believe such argument structure information is better handled in a separate layer of Propbank-style annotation that focuses on the argument structure of each verb. In the treebank annotation, we will avoid using this functional tag. So the above example will be annotated as follows:

```
(VP account
    (PP for
        (NP her disappearance)))
```

The use of **–CLR** in certain context is still kept, since it is more clearly defined. **–CLR** is still used for secondary predicate that is PP or S:

```
(S (NP-SBJ I)
```

```
   (VP described)
        (NP it)
        (PP-CLR as
                   (ADJP sturdy))))
```

[We dropped all instances of PP-CLR from CRAFT.]

```
(S (NP-SBJ uncertainty)
   (VP drives
        (NP-3 people)
        (S-CLR (NP-SBJ *PRO*-3)
                 (ADJP-PRD wild))))
```

4.2 Dropping functional tag **–PUT**

Likewise, we believe the functional tag –PUT adds very little information because it is only used to mark the PP complement of a limited number of verbs such as “put” and “place”. So instead of

```
(VP place
     (NP the book)
     (PP-PUT on
               (NP the table)))
```

we are going to do:

```
(VP place
     (NP the book)
     (PP-LOC on
          (NP the table)))
```

4.3 Expansion of the –LOC tag

Previously, the –LOC tag was only used for locations that could be found on a map. In biomedical texts, we have extended –LOC to refer to more general rather than specific locations.

```
 (S     (NP-SBJ-1 No cataract formation)
                 (VP was
                         (VP observed
```

```
                    (NP-1*)
                    (PP-LOC in
                            (NP the (NML alphaB knockout) lenses ))))
                            .)))

(S      (NP-SBJ (NP Ears)
                (PP of
                        (NP (ADJP Brn3c null) mutants)))
        (VP undergo
                (NP apoptosis)
                (PP-LOC in
                        (NP neonates))
.))
```

4.4 LST

In the biomedical texts, we have encountered multiple list markers within one LST node. We have decided not to show an internal structure for the LST node.

```
(NP     (LST  [i - k])
                two
                (ADJP (NP three week) old)
                 transgenic mice)
```

4.5 Latin terms

Latin terms such as ‘in vivo,’ ‘ex vivo,’ and ‘in vacuo’ should be tagged as flat ADVPs when modifying verbs and ADJP when modifying nouns.

ADVP:

```
(S      (NP-SBJ Both alphaA and alphaB)
        (VP can
                (VP bind
                        (PP to
                                (NP actin))
                        ,
                        (ADVP (ADVP in vitro )
                                and
                                (ADVP in vivo))
                 .)))
```

ADJP

```
(NP (ADJP in vitro) fertilization)
```

Latin abbreviations such as ‘i.e.,’ ‘e.g.,’, ‘etc.’, and ‘et al.’ are also tagged as ADVP. ‘Etc.’ and ‘et al.’ are also typically given the –ETC tag.

```
(S      (NP-SBJ-1
                (NP Sedolisin
        and
                (NP     (NP its several variants
                        [(NP (ADVP e.g.)
                          ,
                          kumamolisin
                          ,
                          aorsin,
                          and
                          physarolisin])
        (VP have
                (VP been
                        (VP found
                                (NP-1 *)
                                (PP-LOC in
                                        (NP archaea)))))
        .)

(S      (NP-SBJ         (NP Vahava
                        (ADVP-ETC et al.)
        (VP have
                (VP demonstrated
                        (NP that mutation)

        .)
```

4.6 Expansion of -TTL

The -TTL function tag was originally used only to mark the titles of created works. But it also marks a form of nominalization, as titles whose internal structure is not nominal can behave nominally. For example, "In the Heat of the Night" or "One Flew Over the Cuckoo's Next" can function as the subject of a sentence or the object of a preposition.

The following is an example of –TTL in its more traditional usage:

```
(NP
 (NP
```

```
  (NP (DT the) (NNP EU) (NN project))
  (NP (NN EUMORPHIA)))
(, ,)
(`` ``)
(S-NOM-TTL
  (NP-SBJ (-NONE- *PRO*))
  (VP (VBG Understanding)
    (NP (JJ human) (JJ molecular)
      (NML (NN physiology) (CC and) (NN pathology)))
    (PP-MNR (IN through)
      (NP
        (NP (VBN integrated) (JJ functional) (NNS genomics))
        (PP-LOC (IN in)
          (NP (DT the) (NN mouse) (NN model)))))))
('' ''))
```

In CRAFT we have extended -TTL to other instances of this referential nominalization that can occur outside of titles. For example:

```
(S
  (S
    (NP-SBJ-1 (NN Significance))
    (VP (VBD was)
      (VP (VBN set)
        (NP-1 (-NONE- *))
        (PP (IN at)
          (S-TTL-FRM
            (NP-SBJ (NN p))
            (VP (SYM <)
              (NP (CD 0.05))))))))
  (CC and)
  (S
    (ADJP-TTL-SBJ (JJ suggestive))
    (VP (VBZ refers)
      (PP (IN to)
        (S-TTL-FRM
          (NP-SBJ (NN p))
          (VP (SYM <)
            (NP (CD 0.63)))))))
  (. .)))
```

This sentence has two nominalizations. "Suggestive" refers to the word suggestive itself rather than the quality of being suggestive. Similarly, the formula (analyzed internally as S because it is read aloud as "P is less than 0.63") is functioning nominally within the larger context of the sentence, in its position as object of a preposition.

See also pp. 52-53 of the 2009 supplementary guidelines for more discussion of -TTL.

Section 5. Addition of –FRM function tag

We have added one new function tag, -FRM, to the top-level constituent (S) of formulas in which a mathematical symbol (<, >, =) is treated as a verb.

```
(S-FRM
        (NP-SBJ (NN p))
        (VP (SYM <)
          (NP (CD 0.05)))
```

The above is interpreted as “p is less than 0.05.” Orthographically, the copula is not realized, thus we’ve created the –FRM tag to denote the difference between formulas and canonical sentence structure.
